# Non-Linear Association Between Phase Angle and Body Fat in a Sample of US Adults

**DOI:** 10.3390/biology14111621

**Published:** 2025-11-19

**Authors:** Federica Frau, Eduardo Pizzo Junior, Stefano Cabras, Myosotis Massidda, Elisabetta Marini

**Affiliations:** 1 Department of Life and Environmental Sciences, University of Cagliari, Cittadella Universitaria Monserrato, 09042 Monserrato, CA, Italy; eduardo.pizzojunior@unica.it; 2 Department of Statistics, Carlos III University of Madrid, 28903 Getafe, Spain; stefano.cabras@uc3m.es; 3 Department of Medical Sciences and Public Health, University of Cagliari, Cittadella Universitaria Monserrato, 09042 Monserrato, CA, Italy; myosotis.massidda@unica.it; 4 Faculty of Physical Education, Gdansk University of Physical Education and Sport, 80-336 Gdañsk, Poland

**Keywords:** body composition, bioelectrical impedance analysis, obesity

## Abstract

Phase angle (PhA) is a widely used bioimpedance index associated with cell mass, membrane integrity, skeletal muscle mass and quality, and body fluid distribution. This study aims to investigate the relationship between PhA and body fat, which is poorly defined in the current literature. The analysis involved 1533 adults of both sexes from the National Health and Nutrition Examination Survey. Anthropometry, bioimpedance analysis, and dual-energy X-ray absorptiometry were used to estimate body mass index (BMI), PhA (at 50 kHz), and body fat, respectively. Non-linear cubic spline regression models were applied to examine the associations between PhA and fat mass (FM), BMI, fat mass index (FMI), and FM%. The relationship between PhA and body fat was non-linear. An initial positive slope was observed with FM, FMI and BMI, followed by a decline, whereas FM% exhibited a flatter initial pattern before decreasing. Men showed more pronounced flection points, at lower fat levels (FM: 23 kg; FMI: 9 kg/m^2^; BMI: 37 kg/m^2^; FM%: 25.5%) than women (FM: 34 kg; FMI: 16 kg/m^2^; BMI: 32 kg/m^2^; FM%: 42.5%). Although most associations were significant, the explained variance of the relationships was lower than 10%. In conclusion, this study demonstrates a weak and non-linear relationship between PhA and body fat. This suggests that PhA variability is predominantly influenced by factors other than fat, a point that warrants consideration in both clinical and routine practice.

## Simple Summary

Phase angle is a widely used index related to body composition. The literature consistently shows its association with body cell mass, skeletal muscle mass in particular, quality, and fluid distribution. However, its relationship with body fat remains unclear, with previous studies yielding conflicting results. This study analysed a sample of 1533 U.S. adults to determine how phase angle changes with different measures of body fat. The results revealed a non-linear pattern: phase angle increased at lower fat levels, then declined as fat levels increased. However, phase angle accounts for only a small proportion of body fat variability. These findings can help to interpret phase angle and better delineate its appropriate application in clinical and routine practice.

## 1. Introduction

The phase angle (PhA, °) is a bioimpedance index that is widely used in biomedical research into body composition [1]. It is calculated directly from the ratio of reactance (Xc, ohms) to resistance (R, ohms), typically measured at a frequency of 50 kHz. Being based on raw data, it does not rely on assumptions about body geometry or constant tissue hydration as inherent in regression equations, thus avoiding a potential source of error. From an electrophysiological perspective, PhA reflects the phase shift induced by cell membranes, which act as capacitors by storing electrical charge and thus delaying the current relative to the voltage. As such, PhA is considered an indicator of both cell membrane integrity and body cell mass [2].

Research has consistently shown that phase angle is associated with health status. Low PhA values have been observed in various clinical conditions, in the presence of inflammation, and are linked to longer hospital stays and higher mortality rates [3]. Furthermore, PhA tends to be higher in men than in women and in physically active individuals, and it declines with age regardless of the population [4,5,6]. It is considered relatively stable across different body sizes and therefore usable as a consistently reliable indicator in diverse populations [7].

Most of these associations can be explained by the relationship between PhA and body composition. Specifically, PhA values are positively associated with body cell mass and quality, particularly reflecting fat-free mass (FFM), skeletal muscle, and the relative amount of intracellular water [3,8,9,10].

The relationship between phase angle and body fat is less clear. In fact, observational studies have revealed conflicting results, and recent reviews have confirmed this inconsistency [11,12,13], with null, negative, and positive associations observed in different samples. Furthermore, some authors have observed a non-linear association between PhA and body mass index (BMI), precisely a positive correlation among individuals with low or normal weight, and a negative association in those with severe obesity [14,15,16,17].

The overall discrepancy among studies is partly attributable to the widespread use of indirect, second-level methods for assessing body fat, such as anthropometry (mainly BMI: e.g., [14,15,16,18,19,20,21]; or skinfolds based estimates: e.g., [22]) or conventional bioelectrical impedance analysis (BIA) (e.g., [17,23,24,25]). Furthermore, most studies have employed statistical approaches that are unable to capture non-linear associations, such as linear correlation [4,14,19,20,21,26] or linear regression analyses [8,15,22].

This study aims to clarify the complex relationship between phase angle and body fat, as indicated by the most commonly employed indices (BMI, FM, FMI and FM%), using validated reference methods for body composition assessment and statistical approaches suitable for modelling curvilinear associations. To minimise the confounding effect of age, the analysis is conducted on a large sample of young adults from the United States.

## 2. Materials and Methods

### 2.1. The Sample

The sample is derived from the National Health and Nutrition Examination Survey (NHANES) [27]. NHANES includes demographic, socioeconomic, dietary and health-related data, as well as the results of physical examinations (medical, physiological and laboratory tests). Data are collected from the non-institutionalised civilian population, primarily through mobile examination centers operating across the United States. The survey protocol is approved by the National Center for Health Statistics (NCHS) Research Ethics Review Board, and written informed consent obtained as the first step.

This study used the 2003–2004 dataset, as it is the most recent to provide data from both BIA and DXA. The entire dataset comprises a stratified sample of 10,122 participants. The analysed sample was selected based on participant age (21–49 years), availability of the variables under study, and data quality. The NHANES methodology [28] involves evaluating the quality of the raw data using the HYDRA modelling programme [29] and fitting the derived impedance and phase angle to a Cole model [30] via iterative non-linear curve-fitting software [31]. In our study, we only used cases that showed an excellent fit to the Cole model (BIDFIT variable = 0), i.e., those with the highest accuracy.

The final sample comprised 1533 adults (810 men and 723 women). Participants were classified into diverse ethnic groups (Mexican American, Other Hispanic, Non-Hispanic White, Non-Hispanic Black, Others, including multiracial), which were pooled to better capture overall variability. This approach follows that of the NHANES growth charts [32]. Furthermore, as reported by Rosa et al. [7], phase angle does not require adjustment for body size and is therefore not influenced by inter-population anthropometric variability that could otherwise bias bioimpedance analysis. Lastly, the sample was not subdivided by age, as the chosen age range of 21–49 years is characterised by relatively stable body composition.

### 2.2. Measurements

The protocols adopted in the 2003–2004 NHANES cycle, including procedures, guidelines, and quality standards, are documented in manuals available on the CDC website [28,33]. All measurements were performed by trained technicians using regularly calibrated equipment. Participants were recommended to fast for six hours before measurements were taken. Body weight was measured with a Toledo electronic scale and height with a Seca electronic stadiometer (Seca, Hamburg, Deutschland). Bioelectrical parameters were obtained using the HYDRA ECF/ICF Bio-Impedance Spectrum Analyzer 4200 and Xitron IS4000 Disposable Electrode (Xitron Technologies, Inc., San Diego, CA, USA). Whole-body DXA scans were performed with a Hologic QDR-4500A fan-beam densitometer (Hologic, Inc., Bedford, MA, USA) and Hologic DOS software (version 8.26:a3*).

The selected variables included demographic data (age and sex), anthropometric measurements (weight, height, and BMI), bioelectrical values (resistance, R, and reactance, Xc), measured at 50 kHz, and DXA measurements (fat mass, FM). Phase angle was calculated as arctan (Xc/R) × 180/π. Fat mass percentage (FM%) was defined as FM divided by weight × 100, and fat mass index (FMI) as FM divided by height squared [34].

The analysed dataset is available online (Supplementary Materials S1).

### 2.3. Statistical Analysis

Descriptive statistics for anthropometric and bioelectrical variables were calculated separately for each sex, and sex differences evaluated by Student *t*-test.

The association between phase angle and fat mass indicators was analysed using multiple non-linear regression. Due to the curvilinear relationship between the variables, the regression analysis was performed using cubic splines, a type of mathematical curve that can be used to interpolate complex relationships in data, with a normally distributed response variable [35]. Cubic splines were estimated as follows:

Y = a + S (x)

where Y is the response (PhA) and S represents the cubic spline term, which is a non-linear function composed of spline coefficients of the corresponding quantitative variable: BMI, FM, FMI, or FM%, each entering in S (x) separately. Although splines cannot be conveniently written in analytical form, they can be interpreted by examining the overall estimated functions showing the direction and strength of the association between the non-linear predictor and the response variable, alongside 95% confidence intervals (shaded areas in the graphs). In this study, the uncertainty surrounding the splines and regression coefficients was based on the assumption of normality of the residuals, which was confirmed by evaluating quantile-quantile plots of the theoretical normal quantiles against the empirical quantiles of the residuals. Notably, splines do not assume a number of flection points a priori, but rely exclusively on raw data, thereby avoiding errors arising from potentially arbitrary or incorrect assumptions. The flection point was defined as the sole point of tangency with a horizontal line drawn on each curve. The confidence interval of the point is defined by the intersections between the tangent line with the boundaries of the shaded area. The significance of the relationship was assessed using the usual F-test. Following the consensus approach based on Bayesian statistics [36], we used *p*-values lower than 0.005 as the significance level. Statistical analyses were performed using the free software R (version 4.5.1) with the MASS library (http://www.R-project.org, accessed on 13 June 2025).

## 3. Results

The sample comprised men and women of a similar age (34.2 ± 8.6 and 35.3 ± 8.4 years, respectively) who exhibited the typical pattern of sexual dimorphism: men were characterised by greater height, weight, and PhA, as well as lower FM, FM%, and FMI than women (*p* ~ 0.000) (Table 1). Both sexes exhibited similar high BMI values, indicative of mean overweight (Table 1).

**Figure 1:**
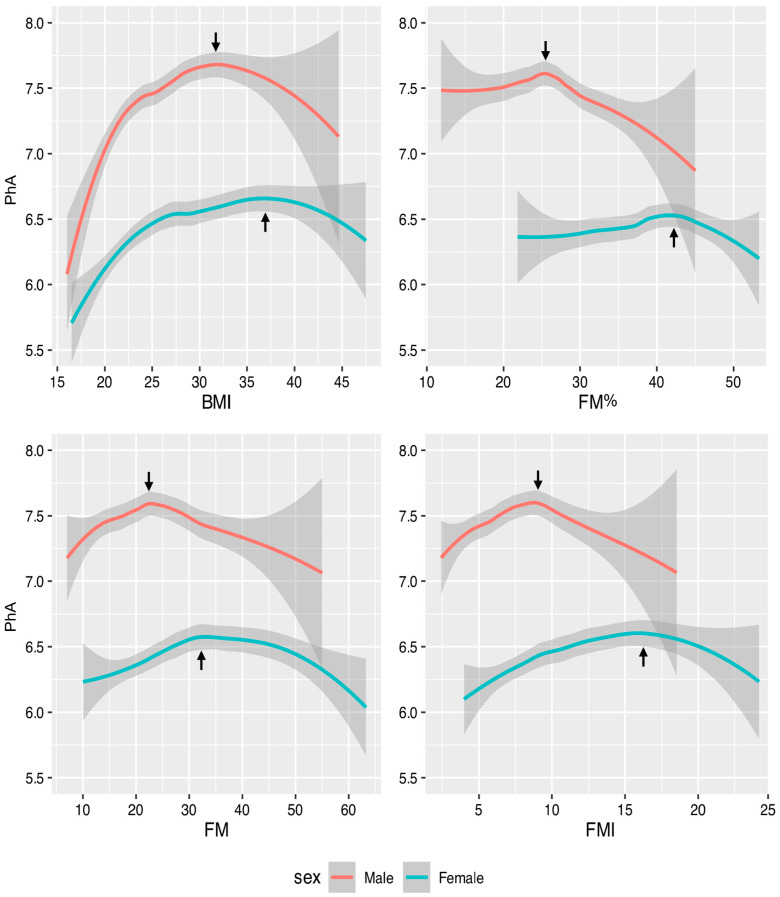
Results of cubic splines regression between PhA values and body fat. Filled areas show the 95% confidence interval about the regression mean estimated with the corresponding spline. The arrows indicate the flection points. The confidence interval of the point is represented by the intersections between the line tangent to the point and the shaded area. PhA: phase angle; BMI: body mass index; FM%: percentage of fat mass; FM: fat mass; FMI: fat mass index.

Regression splines indicated non-linear relationships between PhA and fat mass indices, which were significant for BMI in both sexes and for FM and FMI in women (Figure 1, Table 2). In men, the relationships for FMI and FM% were close to the significance threshold. The amount of variance explained was always small, suggesting the influence of additional causal factors (less than 10%, Table 2). However, including age in the model did not alter the shape of the curve and increased the explained variance by only about 5%.

All the curves were concave. At low or medium fat levels, the association was positive for BMI, FM and FMI, or null for FM%, and at high values it was negative for all indices. Notably, the relationships between PhA and FM or FMI were very similar in shape. The relationship with BMI was also similar, albeit more pronounced, particularly in men. The relationship between PhA and FM% was somewhat different, showing an almost flat trend indicating the absence of association within the range of low and medium FM% values, and a decreasing slope at higher values.

The slopes were sexually dimorphic, with the flection points being more pronounced and occurring at lower fat values in men than in women. The flection points were: 23 kg in men and 34 kg (with a further decrease at 46 kg) in women, for FM; 9 kg/m^2^ in men and 16 kg/m^2^ in women, for FMI; 32 kg/m^2^ in men and 37 kg/m^2^ in women, for BMI; 25.5% in men and 42.5% in women, for FM%.

## 4. Discussion

This study showed that the relationship between PhA and body fat, as derived by DXA, is not linear, with an increasing slope (in the case of FM and FMI, and especially BMI) or a flat trend (FM%), followed by a decreasing slope (all indices). The slopes are sexually dimorphic, with men showing more pronounced flection points, occurring at lower fat values than women.

The observed curvilinear relationships help to explain the inconsistent results reported in the literature. These results are likely to have arisen from the use of different fat indices and from the variable nutritional statuses of the examined samples, as well as the use of linear statistical models to analyse curvilinear relationships. Furthermore, the observed relationships exhibited low explained variance (less than 10%). Bosy-Westphal et al. [14] reported even lower estimates, with only almost 1% of the variance explained for the association between PhA and BMI. These findings suggest that variability in phase angle is largely influenced by factors other than fat or fat-related variables, which influence its values differently as nutritional status varies.

In low/normal/overweight people, the increasing slope is likely related to the effect of the absolute amount of body cell mass, particularly fat-free mass, and a higher intracellular/extracellular water ratio, whose association with phase angle has been consistently observed in the literature [e.g., [8,9,13]]. In our study, this interpretation is supported by the stronger association of PhA with BMI than with FM or FMI, and by the lack of association with FM%. Indeed, BMI includes the amount of FFM, and FM is related to FFM [37,38], whereas FM% is less dependent from the absolute quantity of FFM. The results on FMI, which are similar to those on FM, suggest that height does not adjust for body size as efficiently as weight does. Similar findings emerged in the comparison of height- and weight-adjusted indices of sarcopenic obesity [39].

As fat mass increases, obesity and severe obesity develop alongside their associated clinical complications. This is likely reflected in the onset of phase angle decline. The inversion of this trend along with increasing BMI has also been observed in other studies, with flection points ranging from 30 to 50 kg/m^2^ [15,16,17,24]. Luo and Jin [17] studied the relationship with FM% and identified flection points at 33.5% in men and 38.9% in women (beginning to appear at 16.1% and 18.5%, respectively). Interestingly, in all studies that considered the sexes separately, the flection points occurred at lower values of fat mass in men than in women, consistent with their lower fat mass content and with their lower cut offs for overweight and obesity (men: 25%, women 42%, according to [40]).

The association between obesity and low PhA has already been observed in the literature [23,41], and can be interpreted in various ways. Obesity is often characterised by inflammation, as adipose tissue stimulates the release of inflammatory mediators such as tumour necrosis factor-α and interleukin-6 [42]. In turn, inflammation, along with the associated oxidative stress and decreased membrane quality, has been linked to low PhA values [15,43,44]. Other potential causal factors include the relatively high amount of extracellular water which is common among people with obesity [45,46] and high levels of visceral obesity [20]. Based on these associations, phase angle has been suggested as a marker of increased morbidity risk in people with obesity [12,44].

In summary, both the results of this study and the contrasting findings reported in the literature indicate that phase angle has limited value for informing on body fat. To analyse fat mass variability using raw data, vector analysis, particularly specific bioelectrical impedance vector analysis (BIVA), could provide more information, as it gives details of both phase angle and vector length, with the latter having been shown to accurately estimate FM% [9,41].

Taken together, the findings of this research are bolstered by the following key characteristics: a sizeable sample; DXA-based fat mass assessment; analysis of various fat-related indices; and statistical methods appropriate for non-linear relationships. However, the study is limited by the absence of gold standard techniques to test the association of phase angle with inflammation, extracellular water, or visceral fat within the sample.

## 5. Conclusions

The present study revealed a weak, non-linear association between phase angle and body fat. This suggests that other variables predominantly influence phase angle variability, most likely fat-free mass and the relative content of intracellular fluids in individuals with low, normal or overweight status, and clinical complications in people with obesity. A comprehensive body composition analysis, including fat mass evaluation, should incorporate additional methods, such as bioelectrical impedance vector analysis.

## Figures and Tables

**Table 1:** Sample characteristics by sex with Student *t*-test comparisons.

	Men (*n* = 810) (Mean ± SD)	Women (*n* = 723) (Mean ± SD)	Men vs Women *t*-Test	*p*-Value
Age	34.2 ± 8.6	35.3 ± 8.4	−2.53	0.012
Height (cm)	175.6 ± 7.6	162.6 ± 6.6	35.56	~0.000
Weight (kg)	83.9 ± 15.7	73.6 ± 17.4	12.18	~0.000
R (ohm)	464.0 ± 60.5	562.7 ± 76.9	−28.07	~0.000
Xc (ohm)	60.3 ± 8.2	62.9 ± 8.5	−6.09	~0.000
PhA (°)	7.5 ± 0.8	6.5 ± 0.7	25.91	~0.000
FM (kg)	23.0 ± 8.2	29.7 ± 11.2	−13.46	~0.000
FM%	26.6 ± 5.7	38.8 ± 6.5	−39.15	~0.000
FMI (kg/m^2^)	7.5 ± 2.6	11.2 ± 4.2	−20.97	~0.000
BMI (kg/m^2^)	27.2 ± 4.6	27.9 ± 6.4	−2.48	0.013

R: resistance; Xc: reactance; PhA: phase angle; FM: fat mass, FM%: percentage of fat mass; FMI: fat mass index; BMI: body mass index.

**Table 2:** Cubic splines regression results between PhA and FM-related indices.

	F	*p*	Exp. Variance (%)
BMI (kg/m^2^)			
Men	12.57	~0.000	8.25
Women	13.72	~0.000	7.96
FM (kg)			
Men	2.35	0.0680	1.16
Women	5.04	0.0017	2.50
FMI (kg/m^2^)			
Men	3.35	0.0182	1.54
Women	7.50	0.0001	3.17
FM %			
Men	3.72	0.0146	1.46
Women	1.36	0.3580	0.58

BMI: body mass index; FM: fat mass; FMI: fat mass index; FM%: percentage of fat mass.

## Data Availability

Data derived from public domain resources. Details available on request from the authors.

## Supplementary Materials

The following supporting information can be downloaded at: https://www.mdpi.com/article/10.3390/biology14111621/s1.
