# Supplementary material for: Non-Linear Association Between Phase Angle and Body Fat in a Sample of US Adults
Source: Biology (Basel). 2025 Nov 19;14(11):1621. doi: 10.3390/biology14111621 (PMC12649926; doi:10.3390/biology14111621)
Supplement: Supplementary file 1 [file biology-14-01621-s001.zip › biology-3754470-supplementary.pdf]

SEQN	sex	age	weight	height	BMI	R	Xc	PhA	FM	FM%	FMI
21085	1	26	79.4	172.7	26.6217	491.86	69.56	8.107014	20.7026	25.88077	6.94129
21087	1	28	74.7	169.7	25.93922	523.86	64.04	7.007756	22.608	29.88263	7.850521
21125	1	36	65.8	180.6	20.17392	503.17	63.74	7.261731	11.5194	17.40007	3.531786
21195	1	26	75.4	169.1	26.36842	452.83	61.22	7.749987	20.0175	26.38336	7.000396
21221	1	23	101.5	188.8	28.47489	399.92	59.2	8.485774	27.1473	26.53228	7.615925
21256	1	27	85.1	175.5	27.62965	424.92	62.67	8.454645	22.552	26.30983	7.322018
21286	1	41	76.7	156.3	31.39622	456.42	59.04	7.415229	24.4608	31.6147	10.01273
21346	1	42	74.9	161	28.89549	431.96	59.86	7.943941	23.184	30.6883	8.944099
21437	1	26	101	172.5	33.94245	467.45	57.64	7.068572	36.2598	35.35541	12.18561
21466	1	28	86.8	169.9	30.06997	517.33	74.64	8.270787	27.5199	31.61622	9.53367
21534	1	31	108.2	174.5	35.53337	431.88	50.35	6.68312	41.3311	37.82298	13.57332
21553	1	36	76.9	167.8	27.3113	460.09	67.53	8.41389	20.0891	25.79143	7.134714
21705	1	33	97.5	171.2	33.26573	419.97	52.38	7.149737	30.6946	31.24813	10.4726
21733	1	28	59.9	159	23.69368	542.27	75.73	8.005625	15.7792	26.0492	6.241525
21788	1	30	72.2	170.8	24.74922	456.53	63.84	8.016161	15.7154	21.72451	5.387034
21823	1	33	82.2	177.5	26.09006	510.95	68.09	7.639198	23.8491	28.64196	7.569641
21825	1	27	71.5	164.6	26.39042	524.59	79.43	8.679754	21.9087	30.4598	8.086432
22043	1	32	79.7	176.9	25.46846	443.27	67.28	8.700826	16.1057	19.94388	5.146642
22153	1	48	72.1	173.6	23.92411	453.42	62.35	7.882766	17.5238	24.10068	5.814721
22211	1	32	69.9	173.9	23.11415	539.9	70.02	7.434498	17.7348	25.05439	5.864448
22230	1	23	59.5	177.5	18.88514	549.49	73.81	7.700134	11.6146	19.31575	3.686443
22433	1	44	70.9	160.1	27.66073	440.15	63.51	8.2715	18.8654	26.17181	7.360094
22489	1	41	85.5	174.8	27.98229	547.32	69.82	7.312761	28.2646	33.0678	9.250389
22501	1	37	80.3	166.5	28.9659	411.63	61.01	8.496437	21.2831	26.09761	7.677263
22547	1	28	67.2	171.5	22.84762	482.98	60.59	7.19142	15.5912	22.98424	5.300921
22639	1	31	61.7	157.5	24.87276	459.58	57.16	7.129744	14.5433	23.4714	5.862756
22683	1	38	99.7	169	34.90774	451.8	60.6	7.688989	35.5347	35.71571	12.44169
22687	1	28	113.4	174.7	37.15585	395.43	56.77	8.229854	39.6528	34.31565	12.99236
22836	1	48	90	170	31.14187	423.24	55.95	7.578029	28.3176	31.23649	9.798478
22888	1	48	66.6	155.4	27.5786	487.76	64.04	7.526412	17.4098	25.98369	7.209278
22929	1	39	83.8	168	29.69104	455.45	62.79	7.903012	23.4841	27.82516	8.320614
22972	1	24	80.9	172.9	27.06192	522.47	68.51	7.516843	24.7625	30.45758	8.283321

22976	1	30	66.2	171.5	22.50763	517.8	76.83	8.505731	15.735	23.60245	5.349812
23038	1	42	76.7	162.5	29.04615	437.69	62.84	8.230238	22.0273	28.48215	8.341699
23068	1	41	65.6	165	24.0955	429.45	55.62	7.424398	16.8577	25.14405	6.191993
23220	1	30	69.4	169.3	24.21283	508.67	68.14	7.679074	18.87	27.03791	6.583517
23272	1	23	77.2	162.3	29.3076	465.51	58.23	7.170685	23.0614	29.67417	8.754848
23285	1	47	82	174.6	26.89833	466.24	58.73	7.220933	23.0804	27.70736	7.571028
23408	1	36	72.3	164.4	26.75067	434.75	58.47	7.70968	16.8142	23.03482	6.221177
23455	1	28	72.7	170.6	24.97908	505.15	64.17	7.282065	20.0123	27.21359	6.876049
23456	1	48	56.3	171.4	19.16403	547.85	71.83	7.516005	11.1203	19.52674	3.785253
23477	1	42	77.7	175.6	25.19834	451.06	62.19	7.903675	19.1856	24.442	6.221948
23483	1	39	78.5	168	27.81321	431.52	56.04	7.444577	22.3268	28.32179	7.910573
23502	1	42	75.1	171.4	25.56338	508.13	61.4	6.92686	19.0525	25.07267	6.485304
23550	1	35	101.4	173.3	33.76298	414.53	48.7	6.734663	35.3346	34.44123	11.7653
23561	1	49	81.6	172	27.58248	441.34	56.69	7.36336	19.6666	23.72852	6.647715
23773	1	41	69.1	167.9	24.51188	483.81	64.3	7.618667	17.5617	25.19432	6.229672
23870	1	32	92.4	174.2	30.44918	399.55	58.29	8.363071	22.7742	24.33963	7.504933
24053	1	48	79.1	166.5	28.53304	414.57	53.18	7.353487	24.2754	30.34262	8.756649
24177	1	27	112.9	166.2	40.87257	427.82	51.39	6.885895	51.2193	45.04399	18.54264
24229	1	34	86.7	175.5	28.14912	497.9	76.37	8.792726	25.7818	29.33237	8.370646
24267	1	36	63.2	169.8	21.92006	573.57	76.73	7.668698	18.2093	28.80681	6.315647
24294	1	26	102.4	175.1	33.39855	401.33	58.76	8.393112	33.0823	32.04923	10.79005
24320	1	30	79.9	171.2	27.26084	441.53	62.37	8.097638	21.3878	26.38694	7.297239
24373	1	22	94.8	184.9	27.72902	574.49	68.71	6.85615	36.0357	37.81422	10.54045
24474	1	44	76.5	159.6	30.03279	436	60.81	7.995238	19.8406	25.71598	7.789131
24496	1	36	61.4	170.7	21.07179	461.4	62.79	7.801098	11.0818	17.89222	3.803148
24523	1	23	70.3	158.2	28.08939	489.5	68.79	8.055926	23.3008	32.79106	9.310176
24551	1	41	73.6	162.6	27.83792	447	59.8	7.668961	19.3123	25.87909	7.304541
24616	1	49	88.6	175.2	28.8646	461.48	60.18	7.475533	27.5986	30.99034	8.991225
24637	1	34	75.9	168	26.89201	503.03	62.21	7.089395	23.2994	30.4649	8.255173
24651	1	24	91.9	172.9	30.74153	422.03	66.92	9.089824	28.6707	30.79527	9.590656
24762	1	32	82.2	173.4	27.33843	399.09	61.21	8.792136	19.3529	23.45098	6.436472
24789	1	29	102.6	178.5	32.20112	430.14	56.93	7.587072	33.7421	32.18106	10.58999
24928	1	44	77.7	163.4	29.1016	407.9	52.47	7.37395	24.4711	31.14957	9.165357

24972	1	32	71.9	161.6	27.53253	489	69.33	8.127467	18.721	25.90823	7.168798
24979	1	32	75.1	156.1	30.82011	414.76	51.95	7.180117	21.3269	28.24553	8.752295
24984	1	30	62.8	165.1	23.0391	555.77	69.03	7.120092	17.2596	26.9597	6.331937
25018	1	22	64.5	168.1	22.82571	505.35	71.83	8.148102	15.4793	23.73183	5.477923
25248	1	38	99.6	180.7	30.50303	414.73	43.74	6.045834	30.4767	30.30481	9.333653
25302	1	28	84.9	173.9	28.07427	527.1	67.99	7.394263	28.3575	33.03295	9.377105
25373	1	28	88.7	171.5	30.1575	410.73	64.39	8.986795	24.0283	27.02922	8.169487
25439	1	25	61	165.6	22.24381	504.44	67.58	7.679829	14.5381	23.77348	5.301356
25541	1	46	75	165.7	27.31595	394.93	51.08	7.414359	15.9044	20.91097	5.792583
25588	1	23	88	167.9	31.21629	436.2	58.28	7.659082	25.4142	28.7015	9.015193
25621	1	34	66.5	159.6	26.10693	468.13	71.47	8.751856	18.8028	27.93662	7.381706
25685	1	38	80.3	167.5	28.62107	425.66	61.41	8.270259	21.6212	26.77117	7.706376
25814	1	21	85.2	167.2	30.47664	507.96	62.64	7.069116	28.124	32.88887	10.06015
25842	1	21	53.9	167	19.32662	558.71	85	8.721182	9.7899	18.03958	3.510309
25860	1	45	76.7	161.9	29.26184	483.91	61.9	7.332784	25.2936	32.84904	9.64977
25933	1	39	73.7	161.2	28.36204	453.1	62.47	7.903515	20.0838	26.91542	7.728867
26019	1	45	46.7	156.3	19.11608	563.44	68.98	7.018081	11.5292	24.46925	4.719339
26052	1	41	102.3	177.1	32.61659	419.02	53.38	7.302754	35.1261	34.15929	11.19935
26122	1	28	65.4	163.7	24.40509	457.02	64.47	8.086588	17.2373	26.05739	6.432383
26135	1	45	77	174.7	25.22928	423.23	51.37	6.957865	16.5483	21.51382	5.422101
26187	1	25	61.4	157.8	24.6578	509.42	65.98	7.424705	16.6585	26.74737	6.689935
26229	1	26	82.9	174.3	27.28725	522.58	76.98	8.444384	25.4672	30.3466	8.382748
26370	1	40	56.9	169.4	19.8283	731.61	81.55	6.389799	15.3281	26.82561	5.341479
26394	1	40	108.8	170.8	37.29522	403.49	58	8.240206	44.2744	40.39079	15.17669
26397	1	34	77.6	168.6	27.29899	474.86	71.73	8.659207	22.2213	28.39358	7.817256
26419	1	29	88.8	191.7	24.164	504.75	63.14	7.170858	22.4417	25.02699	6.106769
26443	1	38	86.9	159.6	34.11568	427.31	55.57	7.454872	27.3522	30.92926	10.73808
26588	1	32	68.7	154.1	28.9302	475.82	58.84	7.088802	22.0446	31.94513	9.283184
26613	1	35	88.9	175.2	28.96233	451.7	55.46	7.038379	27.5631	30.91631	8.979659
26649	1	40	68.2	172.8	22.84004	499.61	60.02	6.886645	15.6202	22.826	5.231173
26689	1	38	100.2	175.1	32.68101	383.09	51.73	7.740776	27.5756	27.30111	8.993996
26739	1	43	80.3	173	26.83016	521	59.82	6.581904	23.5278	28.94354	7.861205
26858	1	41	64.8	175.6	21.01483	500.92	64.52	7.383612	14.1852	21.57373	4.600303

26888	1	45	61.6	161.9	23.50104	444.15	59.18	7.638149	12.0382	19.12234	4.592698
26915	1	48	104.4	174.3	34.36416	403.8	50.01	7.099592	37.475	35.73734	12.33522
26955	1	41	78.2	168.9	27.41242	441.64	61.83	8.02553	20.8614	26.48004	7.312808
27018	1	34	63	164.9	23.16857	594.65	77.65	7.485536	17.1016	27.00233	6.2892
27019	1	43	97.2	181.7	29.44126	417.27	54.34	7.465267	30.8302	31.48081	9.338271
27036	1	43	72.5	154.6	30.33326	437.39	63.89	8.373498	27.5761	37.83342	11.53756
27104	1	41	84.6	175.8	27.37364	540.43	57.09	6.055687	25.5216	29.84379	8.25791
27160	1	31	76.5	162.8	28.86374	517.63	77.55	8.588261	21.288	27.56345	8.032044
27161	1	36	73	159.9	28.5513	462.27	56.92	7.058494	20.3452	27.70497	7.957287
27182	1	32	76.5	177.2	24.36318	452.99	57.15	7.232201	18.5462	24.14326	5.906463
27230	1	25	72.9	158.9	28.87219	435.69	59.37	7.811462	21.872	29.46053	8.662449
27239	1	29	64.5	168.3	22.77149	478.53	76.73	9.191764	14.7466	22.69542	5.206234
27251	1	46	66	169.4	22.99943	472.49	54.42	6.602506	17.36	26.0234	6.049548
27285	1	48	83.6	173.3	27.83615	444.07	56.26	7.262584	23.6311	28.10152	7.868407
27288	1	31	83.6	166	30.33822	398.47	53.52	7.699514	20.8385	24.69868	7.562237
27289	1	35	94	174	31.04769	412.17	64.18	8.926191	31.2584	32.9708	10.32448
27307	1	22	78.4	162.7	29.61699	486.2	65.39	7.709731	27.0397	34.3346	10.21473
27313	1	42	71.5	172.3	24.08438	482.1	69.73	8.291353	17.3916	24.27154	5.858265
27341	1	26	89.3	175.2	29.09265	461.54	65.52	8.137807	29.0323	32.06009	9.458303
27348	1	46	79	163	29.7339	429.85	60.41	8.056284	20.6191	25.77887	7.760586
27393	1	44	111.3	176.9	35.56637	431.41	64.01	8.505512	32.0129	28.43501	10.22985
27487	1	38	60.3	168.3	21.2887	557.27	67.83	6.977487	12.6919	20.90378	4.480829
27563	1	24	62.5	164	23.23766	443.25	61.11	7.903262	12.9696	20.55186	4.82213
27582	1	29	68.9	182.5	20.68681	546.37	58.1	6.09582	16.7762	24.24709	5.036953
27793	1	24	89	161.5	34.12282	392.19	57.21	8.362156	25.6087	28.59148	9.81844
27911	1	31	70.8	151.1	31.01018	402.92	59.35	8.443933	22.3557	31.2605	9.791728
27927	1	33	77.4	162	29.49246	507.84	74.18	8.373418	24.8294	31.84395	9.460982
27968	1	28	93.1	181	28.41794	453.17	64.42	8.148964	23.5613	25.101	7.191874
27970	1	22	68.7	165.7	25.02141	484.32	70.4	8.332649	16.9685	24.4298	6.180142
28009	1	48	66.2	160	25.85938	475.92	71.55	8.618239	17.152	25.59202	6.7
28038	1	43	88.7	176.9	28.34444	482.91	58.87	6.988286	27.8604	31.02505	8.902904
28060	1	49	75.3	168.5	26.52132	450.45	42.82	5.449328	20.5262	27.06218	7.229508
28252	1	28	96.8	175.1	31.57207	452.68	65.23	8.260359	30.3753	30.84363	9.90714

28302	1	22	61	173.1	20.35803	530.24	81.8	8.84349	10.8292	17.56017	3.614117
28475	1	24	63.1	164.9	23.20535	527.13	64.91	7.058895	16.4082	25.88163	6.034199
28549	1	34	77.5	177.5	24.59829	509.83	65.85	7.404117	20.7372	26.42671	6.581932
28620	1	42	69.4	177.9	21.92843	473.19	60.36	7.312343	14.6307	20.80568	4.622886
28649	1	49	75.5	169.8	26.18615	456.88	46.89	5.883299	20.7904	27.47306	7.210867
28651	1	28	76.1	180	23.48765	477.94	58.6	7.028572	16.681	21.72526	5.148457
28684	1	28	85.8	186.2	24.74731	486.94	54.36	6.399512	22.1493	25.36631	6.388526
28779	1	22	104.1	184.1	30.71448	485.28	60.44	7.139617	38.9147	36.90567	11.4817
28818	1	21	68.2	166	24.7496	525.89	63.27	6.896771	16.4253	23.81089	5.960698
28819	1	27	109.6	177.5	34.78675	386.37	49.84	7.394648	38.0804	34.46867	12.08662
28860	1	29	63.3	166.4	22.8611	476.31	57.89	6.967175	15.7215	24.54015	5.677895
28879	1	36	75.7	176.8	24.21761	483.42	56.1	6.652442	20.7616	27.1706	6.641961
28938	1	45	90.5	171.9	30.62646	379.43	51.3	7.750479	24.7419	26.93535	8.373004
29140	1	44	93.9	182.6	28.16204	470.34	53.09	6.470587	27.7951	29.34514	8.336173
29199	1	29	99.8	170.7	34.25023	447.34	56.83	7.282538	35.1828	34.96167	12.07434
29202	1	25	93.6	184.8	27.40766	453.71	58.36	7.373604	25.3956	26.85793	7.43626
29302	1	34	94.3	168	33.41128	403.78	60.78	8.628966	30.4741	32.42978	10.79723
29471	1	30	60.8	171.6	20.64757	557.25	69.41	7.140273	13.9838	22.86126	4.748874
29478	1	27	69.2	166.9	24.84239	534.98	65.78	7.04854	19.9113	28.70784	7.148039
29496	1	48	81.2	167.8	28.83846	489.57	59.16	6.927176	29.1167	35.6311	10.3409
29512	1	40	83.4	173.9	27.57826	425.54	53.76	7.242053	19.1077	22.5639	6.318431
29514	1	40	87.7	178.2	27.61749	443.42	58.22	7.526616	25.5587	28.85107	8.048656
29614	1	22	85.7	182	25.87248	498.67	67.42	7.750297	18.061	20.98274	5.452542
29711	1	32	114	185.2	33.23708	433.88	59.28	7.832158	43.3101	37.64659	12.6272
29814	1	41	92.8	177.2	29.55429	476.5	60.62	7.292827	30.3288	32.11171	9.658903
29877	1	27	102.1	178.3	32.11612	404.07	55.71	7.903499	28.6235	27.59721	9.00368
29973	1	42	82	163.6	30.63707	427.99	60.99	8.168981	23.8943	28.69635	8.927456
29984	1	49	87.7	176.2	28.24801	517.38	74.65	8.271095	25.7503	29.00174	8.294123
29988	1	43	91.3	170.8	31.29645	436.12	60.05	7.893141	26.3534	28.88661	9.033601
30028	1	21	67.4	172.4	22.67699	481.94	65.25	7.761227	16.2581	24.10275	5.4701
30058	1	22	82	176.1	26.44205	503.81	62.13	7.069317	22.0873	26.68873	7.12236
30070	1	27	97.3	175.2	31.69893	418.31	66.55	9.119954	30.3632	30.82934	9.891891
30113	1	45	68.4	169.9	23.69569	507.01	67.65	7.648815	16.1311	23.23067	5.588268

30145	1	40	97.6	172.7	32.72391	440.07	51.77	6.743716	32.4662	33.00279	10.88546
30160	1	29	75.3	162.8	28.41098	385.47	57.26	8.515372	18.5343	24.37563	6.993062
30163	1	33	128.9	177.4	40.95865	384.98	48.57	7.232239	46.3439	36.15369	14.72602
30252	1	46	72.2	186.1	20.84704	409.51	53.26	7.455547	8.6973	11.88965	2.51126
30309	1	44	80.5	169.8	27.92033	365.09	51.96	8.158533	19.7748	24.47013	6.85862
30311	1	29	82.2	165.1	30.15627	392.67	57.42	8.382592	23.1133	27.86111	8.479452
30432	1	45	93.5	175.1	30.49575	390.49	54.25	7.964026	27.0752	28.48267	8.830786
30488	1	32	61.1	169.8	21.1917	490.67	68.7	8.026202	11.2967	18.30525	3.918106
30656	1	33	90.6	163.3	33.97472	418.59	65.03	8.905694	26.6729	29.13765	10.00225
30662	1	35	61.8	156.1	25.36195	532.53	78.26	8.424393	17.0557	27.34026	6.999447
30678	1	25	98.5	188.8	27.63327	474.19	57.89	6.998324	29.9411	29.73489	8.3997
30740	1	44	87.7	164.6	32.36979	417.57	58.39	8.015895	24.7142	27.86658	9.121933
30787	1	21	97.8	171.6	33.21271	430.56	59.44	7.913853	33.627	33.9793	11.41967
30815	1	48	88.7	172.8	29.70545	430.29	54.05	7.200743	28.0266	31.48204	9.386052
30961	1	37	78.8	158.2	31.48569	388.64	52.27	7.709884	22.5017	28.19373	8.990884
31035	1	30	78.9	172.7	26.45406	452.76	62.91	7.96516	18.9814	23.76391	6.364196
31082	1	41	86.8	164.9	31.92114	476.57	56.07	6.744453	31.899	36.92044	11.73102
31119	1	23	77.4	172.1	26.1324	477.41	68.63	8.240724	23.7492	30.5122	8.018391
31124	1	44	89.7	173.4	29.83282	402.43	57.27	8.157925	27.4828	30.55916	9.140349
21258	1	26	79.9	169.9	27.67961	487.33	67.28	7.914176	28.5265	35.37482	9.882384
21264	1	41	67.6	157	27.42505	465.18	71.52	8.813519	19.1302	27.95215	7.761045
21838	1	31	62.8	165.6	22.90018	479.02	71.25	8.526564	15.2329	24.05333	5.554717
22111	1	35	61.2	162.7	23.11939	496.02	55.2	6.379443	15.1869	24.48457	5.737121
22348	1	24	69.4	165.8	25.24587	413.09	68.91	9.562698	10.9477	15.58263	3.982482
22577	1	45	91.3	175.2	29.74422	415.73	58.13	8.015522	26.2175	28.75196	8.541282
22709	1	38	63.1	160.3	24.55627	522.7	64.46	7.069369	17.4278	27.35815	6.782277
23095	1	23	87.2	165.2	31.95188	378.67	55.85	8.454835	24.406	27.74673	8.942862
23185	1	30	63.9	164.5	23.61397	527.61	69.27	7.526187	16.5209	25.56168	6.105228
23732	1	34	60.5	172.1	20.42648	533.3	72.1	7.750086	10.677	17.47123	3.604853
23897	1	35	96.1	186.6	27.59943	457.27	61.34	7.68978	23.5943	24.18656	6.776163
24231	1	25	87.8	175.4	28.53878	465.95	55.4	6.815745	29.1326	32.86965	9.469348
25011	1	46	77.8	170.1	26.88877	480.42	59.84	7.140249	21.8206	27.61385	7.541506
25262	1	26	90.5	175.3	29.44996	421.22	65.59	8.926301	20.1267	21.84603	6.549509

25455	1	42	96.8	158	38.77584	356.87	46.22	7.424424	35.6927	36.53662	14.29767
25499	1	37	78.3	177.4	24.88024	437.38	58.75	7.700019	18.6089	23.45927	5.913076
25950	1	26	84.3	170.4	29.03276	446.09	58.73	7.547105	19.8815	23.15432	6.847152
26940	1	27	83.3	176.8	26.64897	411.52	59.37	8.270256	17.6692	20.8966	5.652654
27017	1	39	55.3	165.3	20.23855	653.09	72.91	6.39966	13.0473	23.25987	4.775017
27205	1	34	77.9	166.1	28.23568	490.31	69.61	8.138488	22.497	28.41162	8.154275
27254	1	49	81.5	168.2	28.8075	393.47	53.97	7.862916	25.165	30.41099	8.89498
27550	1	26	77.4	173.7	25.65319	475.23	66.96	8.077081	20.5412	26.15544	6.808104
28641	1	42	85.1	180.4	26.14908	407.84	54.78	7.699722	19.9715	23.19209	6.136739
28702	1	36	80	168.3	28.24371	443.45	59.96	7.751037	19.8013	24.51581	6.990777
28896	1	46	101.5	169.9	35.16246	399.69	47.24	6.775315	36.7337	35.98034	12.72559
29392	1	23	88	168.9	30.84774	466.16	66.43	8.16906	30.3302	33.91782	10.63202
29472	1	39	61.2	170.6	21.02778	578.5	73.8	7.313005	14.0903	22.703	4.841302
29505	1	21	111.2	172.3	37.45711	436.14	54.25	7.130446	42.0045	37.79337	14.14898
29672	1	38	72.9	164.6	26.90716	454.61	56.79	7.161034	15.9544	21.69554	5.888719
29895	1	24	75.9	170.4	26.13982	452.46	67.7	8.577315	17.7584	23.31316	6.11596
29897	1	36	68.9	170.4	23.72903	458.84	61.87	7.729683	17.4644	25.07077	6.014707
30955	1	35	68.8	166.9	24.69879	519.29	72.24	7.974632	16.7633	24.10023	6.017926
31005	1	21	101.8	177.3	32.38399	399.3	62.24	8.935382	22.6871	22.02918	7.21708
21034	1	21	70.3	171.5	23.90161	408.63	54.45	7.638542	13.699	19.19598	4.657583
21067	1	25	108.3	186.4	31.16999	385.06	48.58	7.232226	31.6209	28.94046	9.10086
21105	1	26	75.2	178.1	23.70773	475.81	54.88	6.611856	15.9858	21.02131	5.039721
21109	1	25	104.9	185	30.65011	440.5	55.49	7.221238	31.5969	29.72939	9.232111
21141	1	37	76.3	176.6	24.46488	486.34	62.21	7.332686	21.0643	27.27699	6.754071
21154	1	27	87.9	187.6	24.97602	519.52	61.03	6.734168	24.6351	27.80799	6.999849
21215	1	23	92.1	182.2	27.7436	440.34	55.78	7.261615	27.8616	30.21472	8.392847
21219	1	21	82.5	181.4	25.07145	515.89	69.2	7.689389	22.9183	27.50996	6.964787
21242	1	26	82.4	178.2	25.94847	439.04	62.25	8.127896	20.9872	25.21421	6.609051
21247	1	31	79.8	176.9	25.50041	449.5	58.14	7.414608	23.8233	29.62213	7.612832
21265	1	43	58.2	170.4	20.04397	571.92	31.98	3.205428	16.3698	27.89378	5.637729
21325	1	32	90.9	169.7	31.56459	388.75	54.5	8.036537	25.0519	27.1397	8.699153
21357	1	45	85.5	175.2	27.85466	481.2	66.26	7.893483	24.7919	28.55657	8.076842
21380	1	21	70.8	193	19.00722	517.71	61.09	6.764356	10.7217	15.1005	2.878386

21413	1	23	97.9	172.1	33.05377	444.38	58.27	7.516806	35.7329	36.28361	12.06442
21427	1	35	74.9	181.9	22.63687	480.16	47	5.611187	15.853	21.04974	4.791219
21429	1	41	101.1	172.1	34.13418	461.31	55.83	6.937734	32.2264	31.51677	10.88053
21521	1	34	88.6	181.7	26.83638	438.24	53.81	7.038722	24.13	27.0233	7.308823
21543	1	40	81.6	170.8	27.97141	397.12	50.87	7.343157	23.4986	28.5191	8.055013
21593	1	42	70.6	185	20.6282	526.69	65.98	7.181251	16.3714	23.05104	4.783462
21637	1	43	83.1	181.7	25.17046	535.28	57.68	6.177135	21.3288	25.6525	6.460358
21661	1	40	88.7	169.6	30.83699	416.09	59.66	8.219376	25.8269	28.87723	8.978847
21675	1	31	96.4	180	29.75309	448.79	65.15	8.321739	31.3706	32.18597	9.682284
21690	1	26	88.3	182.5	26.51154	549.47	61.05	6.369195	29.4018	32.93906	8.827713
21704	1	39	87.5	175.2	28.50623	413.54	55.54	7.698945	22.1359	25.42974	7.211556
21723	1	33	71.7	172.3	24.15175	506.43	60.21	6.815411	17.8004	24.61294	5.995967
21724	1	44	92.6	179.8	28.64386	378.39	45.05	6.824927	22.1071	23.62743	6.838367
21740	1	32	78.2	163.7	29.18162	421.58	52.06	7.07892	26.6776	34.07871	9.955186
21741	1	34	85	177.7	26.91808	429.56	66.81	8.915804	22.8252	26.78568	7.228359
21795	1	33	74.3	171.7	25.20277	380.58	56.34	8.486209	14.7616	19.70189	5.007176
21796	1	43	111.9	183.5	33.23211	367.11	47.22	7.373482	35.4234	31.38925	10.52006
21824	1	32	68.3	178.1	21.53242	511.3	67.22	7.536428	13.6226	19.66075	4.294693
21845	1	32	100.8	187.8	28.58047	477.86	64.44	7.730324	30.1564	29.67469	8.550437
21886	1	32	98.8	172.1	33.35763	359.32	50.88	8.117243	30.4087	30.51532	10.26682
22070	1	29	94.6	184.9	27.67052	430.91	56.2	7.476401	17.8794	18.67006	5.229728
22075	1	43	90.2	177.9	28.50064	410.33	54.02	7.546823	26.2425	28.86154	8.291886
22180	1	35	68.1	163.3	25.53729	462.11	66.34	8.229491	13.1462	19.2158	4.929784
22186	1	22	113.7	182.1	34.28788	445.61	59.14	7.607978	38.2415	33.42213	11.53228
22194	1	46	94	181	28.69265	392.47	49.51	7.231515	26.7452	28.13584	8.163731
22234	1	29	95.4	180.9	29.15219	433.01	61.01	8.076923	24.855	25.59556	7.595155
22285	1	46	92.9	177.4	29.51947	377.9	56.07	8.505435	25.2443	26.98569	8.021509
22302	1	40	111.4	179.3	34.65171	413.59	57.83	8.015415	36.3909	32.55183	11.31963
22365	1	49	87.4	186.6	25.10083	506.66	57.73	6.531724	23.086	26.06956	6.630181
22371	1	21	76.9	176.8	24.60151	526.51	68.2	7.425413	23.0375	29.6976	7.370057
22375	1	43	103.9	179.5	32.2468	447.24	53.01	6.794539	37.8687	36.41186	11.75307
22414	1	25	75.2	167	26.96404	513.27	69.03	7.709653	22.2824	29.3469	7.989673
22446	1	25	78.8	180.9	24.07959	495.18	63.96	7.404372	20.1876	25.41156	6.168898

22456	1	49	82.3	185.4	23.9431	440.92	50.47	6.5617	17.4834	20.97885	5.086352
22461	1	43	108.4	178.2	34.1361	398.98	49.62	7.129326	35.2072	32.40683	11.08705
22469	1	46	71.2	184.4	20.93911	461.22	53.85	6.692994	11.4253	15.91336	3.36005
22522	1	30	63.1	163.3	23.6623	470.67	71.35	8.690011	14.0065	22.00896	5.252394
22543	1	47	99.9	180.7	30.59491	496.32	56.37	6.510721	32.3589	32.2348	9.910086
22559	1	44	88.1	176.8	28.18457	451.1	54.91	6.977847	26.1328	29.52308	8.360292
22593	1	25	83.9	183.4	24.94384	508.72	63.54	7.159971	25.8096	30.60965	7.673308
22595	1	44	63.8	172.3	21.49068	567.13	62.01	6.267899	13.6544	21.394	4.599409
22611	1	49	96.7	184.4	28.43837	415.6	48.01	6.62215	21.9584	22.37386	6.457715
22745	1	43	77	186.1	22.233	458.05	41.69	5.217493	17.3177	22.11957	5.000316
22748	1	36	80.1	176.8	25.62524	457.66	49.96	6.257809	22.4546	28.04165	7.183578
22875	1	46	96.7	180.4	29.71347	452.48	56.44	7.150402	27.624	28.24162	8.488159
22952	1	45	90.6	185.3	26.38623	440.02	57.15	7.445377	21.1442	23.08266	6.15801
22982	1	24	92.6	180.3	28.48522	468.28	61.07	7.475929	24.1036	25.79144	7.414647
22997	1	21	56	179.2	17.43862	646.65	80.54	7.139786	9.735	17.26584	3.031517
23000	1	37	61.2	177	19.53462	572.68	69.81	6.987929	13.7892	22.21116	4.401417
23011	1	21	77	186.9	22.04307	450.06	60.85	7.750559	13.5873	17.67111	3.889686
23020	1	22	54.7	177.6	17.34209	534.53	60.52	6.490374	7.6866	13.79383	2.43696
23030	1	36	69.1	171.5	23.49361	405.29	54.44	7.700077	11.611	16.58016	3.947675
23042	1	36	59.6	172.8	19.95992	537.23	63.21	6.744789	12.3404	20.46358	4.132775
23044	1	46	90.8	180.7	27.80799	411.46	56.07	7.811704	25.2481	27.52328	7.732366
23070	1	23	86	192.5	23.20796	529.46	61.64	6.673787	24.1585	27.74466	6.519413
23079	1	48	104	181.6	31.53564	424.64	51.91	7.007659	29.4071	28.02529	8.917036
23110	1	38	106.9	180.2	32.92063	409.06	53.13	7.445531	34.0672	31.53491	10.49124
23204	1	22	96.4	185.7	27.9546	508.02	61.48	6.937387	35.6104	36.70714	10.3265
23210	1	25	77.1	163.7	28.77114	435.04	73.42	9.674489	23.5711	30.11038	8.795945
23242	1	32	86.5	171.5	29.40951	422.37	59.66	8.097166	25.6912	29.46174	8.734864
23256	1	42	72.1	178.7	22.57804	706.84	85.91	6.967315	21.9167	30.15685	6.863191
23305	1	33	91.7	191.9	24.90115	372.25	44.78	6.89592	18.3148	19.77163	4.973387
23306	1	21	66.1	175.6	21.43643	551.94	63.76	6.622154	15.6714	23.57183	5.082282
23309	1	25	82.9	179.1	25.84422	505.85	68.03	7.709418	22.7814	27.17263	7.10214
23314	1	49	93.9	178.1	29.60314	407.81	50.72	7.129585	25.7127	27.10686	8.106247
23338	1	42	87.9	176.8	28.12059	421.11	47.46	6.460632	26.6	30.07084	8.509756

23364	1	26	109.8	183.7	32.53749	385.89	51.15	7.598449	35.318	31.91855	10.46593
23388	1	46	108.3	184	31.98842	445.57	56.84	7.312754	37.9922	34.73919	11.2217
23397	1	21	86.3	170.4	29.72156	407.99	62.65	8.80267	22.8341	26.31669	7.864022
23415	1	46	99.1	173	33.1117	432.7	54.51	7.221579	33.4166	33.45792	11.16529
23438	1	28	107.1	185.8	31.02402	484.7	50.6	5.984396	37.7761	34.93514	10.94273
23454	1	45	96.6	176.4	31.04416	433.62	60.63	8.015325	29.1345	29.90666	9.3629
23488	1	40	84.9	184.4	24.96812	480.58	61.39	7.32276	22.7753	26.62386	6.697956
23495	1	30	61.6	162.3	23.38534	446.66	69.79	8.956926	13.7431	22.03207	5.217322
23520	1	24	88.2	181.1	26.89253	566.56	69.67	7.049247	30.1653	34.05872	9.19752
23553	1	44	83.1	184	24.54513	520.65	66.05	7.272267	24.5835	29.32605	7.261194
23567	1	37	88.7	188.7	24.91035	454.66	62.77	7.914222	23.3919	26.24061	6.569339
23573	1	39	76.6	176.2	24.67272	452.19	59.05	7.485862	23.0327	29.83765	7.418789
23578	1	40	83.6	173.9	27.6444	426.97	46.68	6.26724	26.4658	31.23247	8.751568
23595	1	44	110.7	179.8	34.24272	340.59	46.96	7.903857	32.8276	29.32808	10.15453
23596	1	33	105.4	188.7	29.60035	447.22	57.29	7.343455	31.7432	29.64405	8.914704
23600	1	39	94	183.5	27.91616	479.55	59.05	7.058767	29.8017	31.44817	8.850522
23617	1	48	86.7	167.3	30.97613	459.1	57.59	7.19089	29.3282	33.65286	10.47837
23643	1	31	91.4	179.5	28.36725	407.37	58.56	8.240525	24.9594	26.90982	7.746495
23729	1	21	106.2	184.2	31.30007	402.72	44.03	6.267413	34.0872	32.06967	10.04644
23782	1	31	70.1	172.4	23.58541	518.61	66.99	7.404776	13.651	19.31914	4.592931
23814	1	43	111.4	173.9	36.83715	366.81	43.74	6.83566	41.5101	37.07517	13.72634
23848	1	48	80.9	184	23.89532	550.71	59.73	6.217452	19.0186	23.2572	5.617498
23869	1	44	107	186.3	30.8289	457.89	56.3	7.048393	41.9903	39.03649	12.09827
23894	1	26	71	176.8	22.71401	537.39	67.41	7.190807	18.2558	25.53652	5.840316
23928	1	30	88.2	184.6	25.88244	529.84	62.99	6.815061	27.8968	31.31348	8.186363
24010	1	44	112.5	184	33.22897	359.21	46.14	7.363292	35.2938	31.02833	10.42468
24020	1	42	114.6	185.1	33.44813	396.32	50	7.232141	33.6998	28.98299	9.835909
24029	1	32	108.8	172.2	36.69126	368.66	45.98	7.149667	40.3143	36.63687	13.59543
24035	1	46	99.1	175.4	32.21176	412.59	48.03	6.67324	33.9811	33.81635	11.04532
24044	1	48	79.4	175.9	25.6619	443.83	51.04	6.592299	24.561	30.6723	7.93806
24074	1	31	81.1	181.1	24.72771	507.32	56.28	6.359383	20.2408	24.45513	6.171501
24094	1	25	80	179.6	24.80146	476.85	64.47	7.750304	18.9642	23.43334	5.879249
24117	1	49	92.9	178.2	29.25501	425.12	54.01	7.282919	25.6159	27.19278	8.066669

24124	1	23	50.5	166.8	18.15095	546.62	75.17	7.883188	7.4529	14.63284	2.678756
24141	1	21	103.8	174.8	33.97148	423.43	56.35	7.628781	34.3585	32.86263	11.24479
24162	1	40	110.6	192.2	29.93976	391.24	42.3	6.197834	32.1331	28.57777	8.69853
24183	1	49	70.4	178	22.21942	548.75	59.9	6.257418	17.5632	24.78787	5.543239
24198	1	21	51.8	171.6	17.59119	564.6	73.13	7.425019	7.5894	14.5175	2.577347
24226	1	26	62.4	188	17.65505	598.28	62.99	6.035455	10.3379	16.38346	2.924938
24227	1	31	105.4	172.2	35.54466	458.24	65.38	8.178898	38.0099	35.94585	12.8183
24257	1	22	110.2	181.2	33.56334	415.85	60.74	8.372997	33.9702	30.39602	10.34622
24265	1	39	93	172.2	31.36293	453.63	52.17	6.592679	29.0145	30.8074	9.784729
24278	1	25	92.8	190.1	25.67933	373.96	50.69	7.77034	12.5419	13.42628	3.470556
24292	1	23	79.5	178.1	25.06336	435.66	58.98	7.760683	17.0452	21.22611	5.37371
24301	1	40	82.5	176.2	26.5731	435.33	55.23	7.272761	23.8687	28.8575	7.688063
24441	1	35	101.7	173.6	33.74594	455.37	64.4	8.107077	36.0847	35.13596	11.97357
24447	1	41	81.6	186.3	23.51064	545.78	70.4	7.394314	23.5948	28.73164	6.798148
24492	1	44	97.5	175.7	31.58356	386.08	57.22	8.495979	28.0209	28.4888	9.076921
24502	1	40	79.9	176.4	25.67732	429.04	61.29	8.189072	20.1569	25.05236	6.477786
24576	1	37	132.3	179.5	41.06113	321.66	42.46	7.567036	43.8845	32.59684	13.62016
24603	1	48	85.3	181.3	25.95096	521.11	58.27	6.410006	25.1488	29.25953	7.651061
24609	1	46	82.5	177.7	26.12637	427.28	51.78	6.946921	20.6851	24.75742	6.550625
24626	1	35	96.2	176	31.0563	457.19	68.65	8.607691	25.6917	26.40723	8.294066
24632	1	40	115.9	190.1	32.07149	385.51	54.04	8.035678	32.8242	27.88023	9.083013
24633	1	31	74.8	177.4	23.76809	573.33	66.64	6.663052	22.5287	29.813	7.158613
24659	1	31	93.8	176.7	30.04206	443.54	62.65	8.097131	26.9601	28.59399	8.634723
24672	1	37	83.2	191.7	22.64014	513.46	57.68	6.439639	20.4645	24.47614	5.56874
24688	1	40	90.8	180.5	27.86964	481.92	57.64	6.856333	24.9786	27.33059	7.666792
24730	1	45	98.3	181.3	29.90597	380.84	48.99	7.374078	26.3801	26.4367	8.025661
24746	1	47	70.4	174.6	23.0932	508.41	58.82	6.632142	18.772	26.22942	6.15775
24809	1	37	65.1	162	24.80567	494.78	63.91	7.404565	13.2671	20.28081	5.055289
24824	1	47	99.7	185	29.13075	412.35	53.99	7.505682	30.7648	30.69237	8.988985
24835	1	43	104.8	187.7	29.74629	478.31	56.78	6.80501	31.7336	30.2344	9.007221
24841	1	36	76.9	175	25.1102	483.02	58.97	6.998563	17.1486	22.16942	5.599543
24863	1	33	87.3	173.2	29.10171	443.49	61.93	8.004977	22.0531	24.70346	7.351465
24894	1	29	71.6	178.2	22.54746	449.15	61.69	7.873471	17.3671	23.9182	5.46905

24910	1	32	98.8	186.2	28.4969	419.43	54.25	7.414521	24.5225	24.5078	7.073028
24961	1	30	93.6	178.6	29.34357	431.52	57.88	7.68901	28.5259	30.08671	8.94286
24978	1	33	67.6	176.5	21.69988	579.9	66.99	6.622161	16.133	23.70468	5.178759
24997	1	35	85.9	175.2	27.98498	556.81	62.95	6.480844	33.6629	39.06414	10.96689
24999	1	42	89	169.3	31.05104	410.01	40.56	5.670826	30.5384	33.93823	10.65448
25001	1	45	108.6	178.4	34.12244	380	52.59	7.933456	31.1609	28.45084	9.790848
25023	1	46	87.4	177.8	27.64699	459.98	60.15	7.496172	25.4643	28.71624	8.05505
25060	1	29	82.6	183.1	24.63788	494.29	64.11	7.4351	27.2213	32.64336	8.119554
25119	1	40	62.5	171.5	21.24965	583.54	81.49	8.00528	17.3548	27.43282	5.900535
25150	1	36	65.5	165.5	23.91362	463.94	54.25	6.703178	18.5675	28.18331	6.778872
25198	1	32	102	173.5	33.88451	421.57	55.2	7.506064	39.2693	37.98885	13.0453
25221	1	27	78.5	173.6	26.04775	430.48	54.76	7.292112	20.0665	25.3572	6.658435
25233	1	37	72.2	174.6	23.68365	549.45	66.49	6.936989	22.924	31.21668	7.519724
25239	1	46	82.8	186.9	23.70346	577.38	59.97	5.954087	26.4914	31.5112	7.58379
25266	1	38	72.7	173.5	24.15102	437.16	62.14	8.148425	15.3377	20.95228	5.095201
25289	1	27	76.3	181.6	23.13624	482.25	65.46	7.781201	19.4511	25.20242	5.898105
25311	1	49	87	172	29.40779	401.14	52.81	7.546804	22.5457	25.92679	7.62091
25415	1	28	92.1	176	29.7327	448.24	63.47	8.117097	24.5968	26.49524	7.940599
25416	1	42	98.4	174	32.50099	411.03	56.67	7.903556	33.2729	33.51346	10.98986
25419	1	37	75.2	175	24.5551	457.54	56.02	7.018703	20.4961	27.00949	6.692604
25494	1	21	122.2	177	39.00539	478.26	53.14	6.369427	54.0407	43.48015	17.24942
25553	1	49	73.7	170.6	25.32267	474.31	57.23	6.916786	19.3599	26.17175	6.65189
25576	1	28	85.9	180	26.51235	420.69	61.15	8.332535	18.023	20.80001	5.562654
25580	1	28	81.2	180.2	25.00613	429.48	63.34	8.454306	16.6373	20.3073	5.123577
25608	1	21	60.5	175.6	19.62033	542.9	66.47	7.018571	9.209	15.1046	2.986506
25631	1	41	94.3	173.9	31.18261	411.83	49.76	6.926363	28.6714	30.0517	9.480904
25731	1	36	80.3	174.4	26.40113	456.62	47.51	5.964485	25.6013	31.90742	8.417227
25778	1	36	123.2	188	34.8574	333.2	43.22	7.435713	35.5387	28.68511	10.05509
25788	1	26	68.3	187.4	19.44829	542.58	64.89	6.85578	12.2015	17.68461	3.474354
25844	1	47	105.8	185.2	30.84634	397.32	48.36	6.977321	31.6464	29.71967	9.226614
25859	1	25	89.9	181.3	27.35043	398.09	53.19	7.659344	16.5384	18.20279	5.031505
25943	1	31	83.8	168	29.69104	437.29	56.17	7.363389	23.4337	27.79657	8.302757
26008	1	22	92.3	174.8	30.20778	416.11	61.89	8.526194	24.6899	26.4539	8.080467

26073	1	34	89.8	171.1	30.67442	476.41	48.98	5.893602	34.8203	38.54912	11.89412
26076	1	48	101.9	180.4	31.3113	387.76	52.43	7.751035	30.385	29.59946	9.336545
26089	1	48	103	181.7	31.19804	467.12	59.42	7.292006	35.9743	34.62863	10.89639
26095	1	25	89.6	180.1	27.62362	465.1	57.27	7.058683	26.8249	29.55762	8.270099
26096	1	27	89.1	178	28.12145	429.65	57.48	7.669107	25.0996	28.08045	7.921853
26112	1	46	77.5	169.8	26.87982	436.83	58.91	7.730711	18.0375	23.04093	6.256061
26126	1	24	94.8	169.8	32.88009	481.53	61.43	7.313075	34.4999	36.03066	11.96582
26162	1	47	97.6	172.3	32.87602	400.49	50.95	7.292818	27.8211	28.38031	9.371384
26191	1	49	92.2	182.8	27.59171	455.52	48.2	6.065721	28.238	30.52262	8.450483
26292	1	43	65.6	168.9	22.99559	446.85	51.94	6.663203	11.7036	17.7671	4.102609
26297	1	22	70.6	174.4	23.21196	399.58	54.17	7.771377	10.3561	14.40646	3.404891
26299	1	27	81.4	185.8	23.57941	480.71	60.81	7.251614	19.1309	23.42805	5.541712
26344	1	37	82	181.5	24.89205	401.4	50.21	7.170604	18.0503	21.90235	5.479377
26358	1	46	84.4	189.6	23.47825	533.32	60.95	6.551318	23.8991	27.83354	6.648212
26407	1	30	62.4	171.7	21.16625	489.99	65.29	7.638398	12.5373	19.88715	4.252687
26441	1	27	84.9	175.8	27.47071	431.14	58.37	7.760938	22.1245	25.85033	7.158726
26481	1	33	115.4	189.7	32.06795	384.26	49.84	7.435252	38.6328	33.40016	10.73548
26507	1	21	122.2	198.3	31.07605	399.03	48.71	6.997702	37.4143	30.30806	9.514636
26561	1	41	80.9	177.1	25.79357	541.95	61.46	6.500941	23.7736	29.19736	7.579801
26592	1	27	69.9	178.4	21.96279	603.9	69.66	6.612433	19.0054	27.04182	5.971553
26620	1	37	110.8	183.5	32.90543	447.17	54.51	6.987895	37.5846	33.68185	11.16189
26661	1	41	88.3	177.9	27.9003	468.2	58.32	7.140506	27.0576	30.34333	8.549434
26667	1	28	110	179.3	34.21623	375.81	50.14	7.648193	37.5303	33.8116	11.67405
26687	1	26	58.5	182	17.66091	596.66	75.27	7.231658	8.6171	14.65398	2.601467
26720	1	35	75	174	24.7721	530.8	78.38	8.46481	19.8649	26.19048	6.56127
26723	1	49	93.2	187.8	26.42559	479.09	54.84	6.561803	30.1596	32.26847	8.551344
26771	1	25	81.9	179.1	25.53246	505.33	67.34	7.639077	24.2529	29.21023	7.560883
26778	1	36	80.1	168.2	28.31265	530.74	76.01	8.209785	27.7321	34.26423	9.802363
26821	1	26	78.6	182.3	23.65098	412.59	55.86	7.761132	16.0579	20.41426	4.831872
26829	1	24	69.9	180.3	21.50234	535.24	59.09	6.328609	13.0144	18.4142	4.003434
26835	1	45	76.4	171.4	26.00589	487.98	56.38	6.62317	22.5877	29.00284	7.688655
26837	1	29	62.9	183.2	18.7413	536.73	59.26	6.329197	13.6951	21.50163	4.080508
26880	1	42	75.3	176.8	24.08965	432.1	53.21	7.059141	15.6485	20.61036	5.0062

26901	1	29	89.8	184.3	26.43782	442.96	51.02	6.602658	27.1906	29.81036	8.005125
26902	1	42	103	183.7	30.52242	406.38	56.82	8.015152	28.6812	27.72113	8.499219
26909	1	31	77.7	181.6	23.56076	562.1	61.66	6.288293	20.9404	26.70298	6.349701
26950	1	26	85.7	184.8	25.0944	426.77	57.02	7.659073	18.4862	21.47052	5.413071
26976	1	21	77.8	184.7	22.80583	462.42	64.74	8.025626	13.5437	17.29452	3.970119
27028	1	22	76.3	174.5	25.05727	432	54.8	7.271762	14.765	19.12629	4.848893
27048	1	33	70	161.4	26.87144	434.66	54.14	7.140217	18.7883	26.82648	7.21241
27057	1	46	82.6	169.2	28.85223	453.53	59.95	7.577501	23.8576	28.69704	8.333473
27124	1	28	74.7	174.6	24.50373	568.79	69.74	7.028665	21.2733	28.2544	6.978248
27175	1	44	98.8	175.7	32.00468	381.91	52.04	7.811224	28.1602	28.44091	9.122045
27193	1	21	78.2	179.1	24.37898	517.48	65.56	7.262535	18.1486	22.99617	5.657857
27204	1	39	76.6	166.2	27.73108	413.76	58.74	8.138199	20.0964	25.85707	7.275389
27228	1	26	113.3	183.6	33.61124	367.07	55.91	8.731391	33.3599	29.45653	9.896449
27243	1	34	90.7	178.7	28.40261	406.41	56.18	7.924287	24.7838	26.89016	7.76102
27332	1	25	82.1	177.8	25.97046	524.64	66.74	7.292353	24.714	29.94994	7.81771
27382	1	28	92.1	183.7	27.29237	461.3	55.33	6.87575	26.9304	29.00067	7.980397
27387	1	30	80	188.8	22.44326	437.45	56.27	7.3738	15.7404	19.50454	4.415824
27436	1	25	79.3	176.7	25.39803	456.45	71.48	8.977061	19.0163	23.94985	6.0905
27442	1	22	90.2	173.8	29.86118	437.05	54.44	7.14052	29.0483	31.73114	9.616592
27448	1	22	114.8	186.3	33.07624	412.32	47.34	6.581679	37.6502	32.76463	10.8478
27468	1	28	102	169.2	35.62866	407.74	51.87	7.292489	38.1282	36.99831	13.3182
27469	1	29	86.4	192.6	23.2917	464.85	50.74	6.257206	20.4905	23.57528	5.523826
27471	1	47	91	166.6	32.78622	435.61	49.25	6.481138	31.7918	34.80025	11.45421
27478	1	22	116.2	173.2	38.73561	442.31	64.53	8.363302	44.0097	37.4932	14.67076
27544	1	43	50.3	174.2	16.57569	578.29	52.73	5.227029	8.5769	16.91399	2.826403
27554	1	37	101.2	185.4	29.44158	390.54	51.35	7.537334	29.3864	28.95198	8.549229
27595	1	46	130	184.7	38.10742	394.18	47.63	6.926739	51.8872	39.70758	15.2099
27606	1	21	72.3	173.5	24.01814	396	54.46	7.883613	10.2021	14.05271	3.389149
27660	1	45	109.5	186.5	31.48157	363.16	45.88	7.242162	30.3446	27.47013	8.724162
27676	1	35	90	178.9	28.12042	480.06	57.84	6.90678	30.1067	33.0025	9.406812
27693	1	32	56.9	159.8	22.28223	512.57	75.32	8.423644	11.1718	19.38219	4.374915
27701	1	26	86.5	181.7	26.2003	460.6	63.91	7.954039	24.8803	28.28863	7.536084
27712	1	39	95.5	180.1	29.44259	389.18	53.59	7.893618	25.1366	26.10735	7.749597

27723	1	44	82.8	180.5	25.41417	464.07	62.5	7.720393	18.0761	21.63295	5.548177
27725	1	24	82	175.8	26.53237	483.33	53.79	6.379706	24.9259	30.01645	8.065162
27743	1	26	72.6	182.8	21.72622	428.03	57.11	7.64858	10.2666	14.13965	3.072375
27747	1	39	103.2	179.3	32.10104	382.32	50.94	7.637914	29.867	28.97992	9.290327
27806	1	25	90.6	189	25.36323	566.07	67.8	6.865978	28.3417	31.09272	7.934184
27847	1	34	90.9	183	27.14324	416.2	54.06	7.445894	22.4077	24.15516	6.691063
27893	1	35	71.3	180.3	21.933	476.15	58.97	7.09954	12.7614	17.64245	3.925607
27921	1	49	78.9	164.3	29.22819	482.06	68.01	8.087505	24.3083	30.58256	9.004912
27941	1	25	108.5	167.8	38.53415	401.33	54.19	7.740346	42.5285	38.89878	15.10415
27967	1	21	58.3	179.7	18.05396	597.87	76.7	7.354133	9.5315	16.25468	2.951652
28077	1	44	88.7	180.9	27.10482	464.11	65.56	8.097685	24.5108	27.18034	7.489975
28084	1	21	67.4	179.5	20.91852	496.75	64.78	7.475598	13.1521	19.48174	4.081936
28094	1	35	104.1	181.6	31.56596	424.71	51.4	6.937668	33.8968	32.33181	10.27844
28124	1	43	82.9	177.4	26.34191	451.73	55.71	7.069636	19.9518	23.96757	6.33979
28156	1	30	97.8	179.8	30.25238	430.45	62.8	8.363341	29.0261	29.23145	8.978614
28158	1	45	87.8	167.1	31.44428	436.33	64.51	8.475295	29.9907	33.97937	10.74073
28173	1	22	120.7	186.8	34.59024	391.39	49.3	7.220712	43.1419	35.52072	12.36362
28213	1	42	87.8	181.9	26.53561	429.61	54.04	7.210806	24.7575	28.00252	7.482408
28230	1	33	69.1	188.1	19.52992	561.66	62.21	6.349354	10.0626	14.43996	2.84402
28239	1	25	80.8	188.3	22.78824	513.18	63.83	7.130139	18.5401	22.71943	5.228913
28260	1	46	64.2	167.3	22.93734	513	63.9	7.140463	16.5373	25.74263	5.908439
28267	1	26	64.3	183.6	19.07505	503.19	56.89	6.481071	12.1847	18.67367	3.614677
28310	1	30	100	179.5	31.03638	436.11	63.93	8.403332	27.7582	27.52053	8.615141
28324	1	43	78.3	184.4	23.02714	487.79	58.34	6.856088	14.6173	18.40673	4.298781
28362	1	33	97.9	179.3	30.45244	471.13	62.36	7.587666	37.2265	37.65376	11.57955
28389	1	24	70.4	181.5	21.37073	583.39	70.29	6.906809	18.0893	25.34332	5.491216
28405	1	41	115.1	176.7	36.86398	364.67	47.23	7.42439	36.5264	31.68425	11.6986
28413	1	25	86.5	189.3	24.13876	490.82	64.71	7.557741	20.48	23.48538	5.715164
28428	1	24	75.2	181.4	22.853	448.65	62.57	7.994685	12.2245	16.12206	3.714981
28440	1	26	71.4	172.4	24.0228	495.85	79.69	9.2129	14.9271	20.66095	5.02228
28447	1	35	89.6	185.8	25.95473	462.28	62.59	7.761447	21.6984	24.00502	6.285449
28457	1	41	90.2	186.3	25.98848	498.34	56.87	6.541846	25.8241	28.26006	7.440455
28495	1	47	68.1	178.1	21.46937	426.87	52.19	7.008652	9.9814	14.46186	3.14676

28511	1	40	99.2	180.9	30.31339	487.04	66.63	7.842383	33.1651	33.3183	10.13454
28563	1	28	82.8	173	27.66547	453.16	62.24	7.873374	26.985	32.27933	9.016339
28583	1	28	116	178.1	36.57044	426.01	50.04	6.733492	41.6127	35.58539	13.11892
28597	1	21	91.8	181.9	27.74452	387.66	58.35	8.628449	22.6212	24.51424	6.836759
28667	1	36	77.3	176.3	24.86995	421.04	59.25	8.066922	20.2307	26.0945	6.50888
28698	1	43	59.9	178	18.90544	536.75	60.87	6.500909	13.0881	21.69095	4.130823
28731	1	25	71.7	180	22.12963	473.54	67.39	8.157961	12.3576	17.01799	3.814074
28833	1	49	68.4	172.1	23.09374	463.75	61.88	7.649081	15.3528	22.43141	5.183533
28862	1	28	76.5	186.8	21.92339	523.16	60.62	6.642388	19.9836	25.9339	5.726905
28870	1	49	105.2	190.2	29.08002	417.7	48.85	6.704138	30.6133	28.86051	8.462314
28924	1	27	68.8	180.9	21.0238	491.68	70.15	8.17877	12.5337	18.16147	3.83003
28928	1	41	72.8	172.2	24.55077	506.69	52.54	5.944161	17.6337	24.0523	5.946715
28973	1	28	79.9	175.5	25.94135	559.07	69.14	7.089344	27.602	34.29655	8.961616
29000	1	43	91	180	28.08642	412.91	53.48	7.424699	24.2016	26.24487	7.46963
29033	1	33	73	187.4	20.78661	497.81	65.18	7.505741	10.1157	13.77303	2.880426
29061	1	25	80.9	183	24.15719	423.19	53.24	7.21183	16.4591	20.16461	4.914778
29095	1	32	84	172.9	28.0989	427.56	64.43	8.638412	22.4567	26.33435	7.512006
29097	1	48	90.6	165.6	33.03753	348.11	40.96	6.745068	23.9635	26.32079	8.738353
29146	1	32	82.5	177.5	26.18528	513	73.65	8.22997	23.0665	27.60911	7.321246
29161	1	46	60	171.8	20.32848	531.24	65.32	7.048525	12.9092	21.39711	4.37374
29163	1	46	90.8	173.3	30.23352	451.65	54.02	6.856389	28.5674	31.10142	9.512038
29201	1	29	91.4	173.1	30.50367	435.96	59.95	7.882889	24.0498	26.16298	8.026336
29240	1	23	91.6	192.6	24.69351	472.08	67.27	8.16862	19.6126	21.43283	5.287162
29334	1	41	97.5	182	29.43485	509.36	59.21	6.663664	37.138	37.72565	11.21181
29368	1	34	81.2	169.6	28.22957	507.14	75.25	8.505924	21.8789	26.56522	7.606306
29424	1	29	78.8	168.3	27.82006	432.22	48.86	6.480246	24.4369	30.49261	8.627359
29431	1	26	92.7	178.4	29.12662	441.73	59.88	7.770836	33.1241	35.41238	10.40769
29434	1	32	76.4	182.4	22.9638	405.97	46.76	6.602728	16.471	21.50132	4.950742
29452	1	36	104.8	172.6	35.17869	403.77	55.38	7.86252	38.2503	36.32555	12.83965
29484	1	32	70	168.4	24.6839	465.66	63.04	7.760508	11.9261	16.82802	4.205467
29494	1	32	70.1	185.1	20.45998	455.3	56.95	7.170327	9.8494	13.8051	2.874729
29547	1	28	69.7	174.9	22.78522	579.82	78.6	7.770916	19.6441	27.84067	6.421737
29597	1	30	84.2	179.1	26.24949	572.67	70.72	7.079143	27.1408	31.96691	8.46119

29598	1	24	62	179.5	19.24256	531.47	74.6	8.046424	8.3725	13.51951	2.598521
29613	1	28	75.8	171.2	25.86197	464.49	67.1	8.281119	19.9442	26.22018	6.804702
29647	1	43	66.8	173	22.31949	534.66	67.35	7.22109	17.8648	26.51457	5.96906
29651	1	26	70.4	163.4	26.36748	419.18	68.34	9.345817	16.9764	24.03564	6.358307
29723	1	45	89.8	180	27.71605	436.64	47.9	6.288613	28.4084	31.43051	8.768025
29772	1	24	102.8	184.2	30.29799	386.17	49.4	7.333162	27.4789	26.31869	8.098789
29786	1	25	69.6	184.2	20.51304	494.71	63.37	7.34304	13.6889	19.38334	4.034496
29793	1	40	84.5	169.6	29.37684	418.26	65.95	9.038811	24.5552	29.01573	8.536735
29802	1	28	69.8	171.2	23.81485	458.63	66.33	8.290685	13.9142	19.6227	4.747344
29827	1	33	65.1	175.4	21.1603	541.74	81.74	8.649412	12.5681	19.01696	4.085173
29844	1	28	58.9	181.5	17.87977	609.49	53.75	5.055391	12.2109	20.78522	3.70676
29853	1	42	78.5	174.9	25.66197	487.17	58.95	6.936592	20.6786	26.03712	6.759919
29861	1	48	107.5	178.1	33.89071	436.9	47.54	6.237635	36.7364	33.75974	11.58161
29871	1	40	83.6	172.4	28.12754	465.27	53.83	6.63227	22.6866	26.92807	7.632993
29879	1	40	77.2	172.4	25.97424	496.96	65.69	7.577408	24.0198	30.77028	8.081554
29884	1	39	88.2	171	30.16313	441.57	60.65	7.873614	24.2069	27.36996	8.27841
29885	1	48	81.4	167.9	28.87507	408.33	55.72	7.822448	22.2981	27.10705	7.909818
29928	1	37	59.8	176	19.30527	532.83	54.5	5.863416	10.3383	17.12822	3.337519
29939	1	30	86.6	183.5	25.71851	434.74	62.26	8.209607	22.2274	25.37485	6.601103
29956	1	27	111.7	179.3	34.74502	351.03	52.65	8.597991	30.8381	27.91958	9.592395
29985	1	47	74.3	174.1	24.51271	451.95	60.62	7.688974	14.7764	19.5379	4.87496
29993	1	44	94.9	187	27.13832	372.8	46.76	7.190208	21.0013	22.1278	6.005691
30019	1	44	67.5	173.3	22.47536	525.4	66.28	7.231615	15.7472	23.07323	5.243318
30055	1	42	77	187.4	21.9256	531.37	56.88	6.136283	14.5405	18.89159	4.14038
30069	1	34	77.3	171.3	26.34297	437.18	56.86	7.455717	21.3319	27.19775	7.269672
30071	1	47	80.9	170.8	27.73146	486.67	52.7	6.207531	30.0637	36.75813	10.30544
30083	1	33	78.5	173.9	25.95796	399.52	61.64	8.844371	17.841	22.54602	5.899566
30096	1	47	71.2	174.1	23.48997	491.67	54.54	6.358933	17.0222	23.60169	5.615884
30217	1	36	73.1	176.4	23.49201	499.59	60.63	6.956915	19.2792	26.28449	6.195721
30257	1	41	72.5	176.2	23.35211	472.18	57.48	6.978338	18.8482	25.69839	6.07097
30267	1	42	89	172.9	29.77145	429	53.13	7.099461	24.4676	27.26493	8.184674
30314	1	39	125.7	185	36.72754	353.8	46.96	7.608747	43.741	34.56095	12.78042
30315	1	35	68.6	179	21.41007	567.18	74.97	7.577212	18.3731	26.41514	5.734247

30372	1	33	103.4	177.4	32.8559	397.49	52.89	7.627641	37.7322	36.22144	11.98961
30373	1	25	80.9	177.9	25.56211	440.55	60.43	7.863217	20.1373	24.87748	6.362816
30412	1	39	75.2	177.5	23.86828	473.98	60.05	7.262662	15.8995	20.89798	5.046459
30465	1	31	71.9	176	23.21152	438.04	56.19	7.353399	11.5892	16.01678	3.741348
30489	1	36	95.6	178	30.17296	537.73	69.84	7.445311	30.7826	32.06177	9.715503
30499	1	25	76.9	178.1	24.24368	503.26	60.81	6.926685	20.7671	26.66198	6.547086
30526	1	43	94.3	185.5	27.40463	469.12	55.03	6.724476	23.993	25.27359	6.972632
30534	1	37	64.6	175.5	20.97386	450.05	61.17	7.791491	12.3458	19.0036	4.008344
30556	1	41	117.7	181.4	35.7686	343.81	43.01	7.171232	39.4417	33.34001	11.98619
30603	1	39	86.3	172.4	29.03597	406.82	60.07	8.464439	22.5763	25.88129	7.595883
30624	1	41	73.3	174.8	23.9895	514.43	72.94	8.127974	18.5882	25.26267	6.083514
30672	1	27	79.8	183.1	23.8027	441.61	62.85	8.15848	18.0978	22.45633	5.398202
30697	1	33	93.5	178.6	29.31222	423.88	64.11	8.670132	23.7819	25.11203	7.455618
30733	1	22	76.8	179	23.96929	500.74	58.29	6.673054	18.8743	24.45304	5.890671
30738	1	46	99	182	29.88769	453.69	55.95	7.069419	31.4044	31.48895	9.48086
30746	1	22	70.6	173.2	23.53471	532.27	64.13	6.906724	17.6381	24.80718	5.879712
30767	1	24	56.2	166.1	20.37028	534.29	71.67	7.689591	11.9733	20.96955	4.339849
30795	1	40	90.5	175.3	29.44996	423.8	63.49	8.587905	29.3606	32.17329	9.554349
30798	1	39	83.9	168.4	29.58542	462.24	55.2	6.845646	27.5906	32.39451	9.729196
30856	1	23	83.8	183.9	24.77882	440.76	58.5	7.608456	20.9036	24.61094	6.180984
30899	1	39	78.7	186.1	22.72385	478.83	57.77	6.916142	17.6207	22.20896	5.087804
30926	1	49	81.3	172.3	27.38546	460.53	59.32	7.383905	23.6205	29.00491	7.956435
30932	1	24	105.8	180.3	32.54574	379.68	54.04	8.159067	31.2328	29.38487	9.607701
30936	1	30	98.2	184.9	28.72352	413.42	51.64	7.160405	24.2268	24.24384	7.086344
30987	1	23	70.9	180.4	21.78578	445.51	60.31	7.760232	13.9588	19.64495	4.289187
31024	1	39	69.1	185.2	20.14634	508.51	54.52	6.146094	14.6168	20.97141	4.261577
31044	1	43	79.2	168.6	27.86186	460.31	63.63	7.924181	23.5688	29.49278	8.291294
31065	1	21	82.2	179.1	25.62599	495.22	74.63	8.638894	19.2119	23.18248	5.989342
31066	1	32	102.2	184.7	29.9583	399	60.56	8.700733	27.0231	26.08319	7.92139
31111	1	34	100	184.4	29.40886	425.65	53.62	7.221327	31.98	31.56754	9.404953
21153	1	49	78.1	169.9	27.05604	419.78	62.14	8.485792	20.7276	26.11621	7.180625
21193	1	40	109.7	178	34.62315	374.17	56.45	8.648441	29.4056	26.57502	9.280899
21239	1	38	84.3	176.9	26.93841	435.62	48.25	6.349395	20.6739	24.44304	6.606429

21271	1	45	114.1	179	35.61062	449.74	58.01	7.394081	39.8066	34.69529	12.42364
21272	1	44	125.8	187.2	35.89789	355.34	45.77	7.383796	38.2347	30.18009	10.91053
21418	1	22	69.6	182.4	20.9199	516	65.37	7.262257	10.5768	15.00215	3.179103
21432	1	30	86.2	176	27.828	370.38	52.58	8.137967	22.6428	26.01607	7.309788
21441	1	37	78.5	173.7	26.01777	540.74	69.94	7.414468	22.5968	28.40121	7.489405
21465	1	41	76.1	175.6	24.67946	435.51	59.04	7.771253	14.5956	19.3474	4.733397
21472	1	45	77.8	183.5	23.10508	479.22	64.88	7.761019	17.6645	22.58619	5.246011
21491	1	31	66.1	161.3	25.40579	435.18	59.85	7.883845	16.3377	24.02705	6.279458
21495	1	21	83.3	175.6	27.01444	515.92	65.54	7.282273	23.9148	28.57416	7.755642
21513	1	22	86.3	167.5	30.75963	395.97	60.59	8.771655	24.5822	27.91804	8.761755
21583	1	43	91.9	178.4	28.87525	437.49	54.49	7.13989	24.4331	26.15994	7.676953
21646	1	29	75.6	172.3	25.46544	506.58	70.11	7.933682	19.5793	25.56495	6.595179
21652	1	42	102.9	171.8	34.86335	438.1	52.09	6.815912	35.7623	34.57362	12.11655
21774	1	26	69.3	168	24.55357	453.38	68.16	8.618071	14.5553	20.67523	5.157065
21783	1	37	98.6	188.8	27.66132	479.35	58.09	6.946907	24.8933	24.87765	6.983586
21842	1	35	93.9	183.8	27.79551	426.85	56.12	7.53677	20.4698	21.47579	6.059302
21852	1	40	101.2	176	32.67045	483.3	62.6	7.425067	29.4057	28.97584	9.493059
21855	1	29	92	173.1	30.70391	449.04	62.71	8.005614	24.3462	26.08542	8.125256
21928	1	40	95.2	172.6	31.95622	398.76	62.02	8.915856	24.993	25.94918	8.389514
21952	1	21	57.9	174.6	18.99285	581.35	80.46	7.933872	8.5727	14.68207	2.81209
21957	1	42	85.5	165.6	31.1778	411.3	57.22	7.975024	25.1672	29.2808	9.177285
21995	1	32	67.9	170.8	23.27523	521.86	80.69	8.863568	11.9798	17.47014	4.106519
22163	1	27	81.7	169.8	28.33653	385.39	55.95	8.322284	18.6659	22.72364	6.474013
22264	1	47	105.4	177.5	33.45368	469.06	60.92	7.445165	35.2958	33.22083	11.20279
22391	1	29	63.7	176.3	20.49438	478.45	60.61	7.261905	8.4043	12.993	2.703939
22694	1	42	86	174.5	28.24279	422.19	57.53	7.811407	21.4743	24.72064	7.052257
22886	1	23	84	180	25.92593	451.59	58.81	7.465342	20.1342	23.7176	6.214259
22925	1	30	70.2	166.4	25.35306	475.89	72.74	8.762128	16.3592	22.80826	5.908203
22950	1	42	62.5	161.1	24.0818	627.38	81.82	7.476041	18.8494	29.85826	7.26284
22957	1	33	84.9	174.7	27.81774	485.58	58.85	6.9475	24.6536	28.78829	8.077827
22981	1	40	112.1	171.7	38.02463	408.18	52.8	7.415237	41.3533	36.52654	14.02715
23053	1	25	74.3	175.6	24.09571	455.33	74.4	9.366763	14.4095	19.20578	4.673044
23132	1	26	80.8	177	25.7908	477.32	59.37	7.130176	20.6238	25.30124	6.582974

23155	1	28	76.7	179.3	23.85804	527.51	72.45	7.873187	14.0401	18.03558	4.367266
23434	1	21	98	178.7	30.68859	499.27	66.97	7.689316	34.4692	34.6848	10.79399
23484	1	29	90.8	173.2	30.26844	431.95	62.71	8.322354	19.4215	21.16593	6.474213
23768	1	33	92.8	178.1	29.25635	483.45	73.2	8.679653	27.5161	29.26429	8.674791
23811	1	38	77.6	164.8	28.57244	436.92	68.58	8.997843	19.9405	25.59497	7.342123
23859	1	47	74.4	174.6	24.40532	562.72	61.43	6.257935	20.5057	27.31569	6.726453
23935	1	31	104	173.4	34.58877	330.88	56.92	9.861369	23.6027	22.48519	7.849889
23936	1	44	73.7	179.4	22.89932	530.61	55.96	6.04568	20.508	27.76161	6.372039
24025	1	26	93.4	170.6	32.09141	465.7	60.4	7.434873	35.8387	38.05741	12.31386
24077	1	22	80.7	180	24.90741	370.9	50.28	7.771078	12.2193	15.01877	3.771389
24153	1	28	59.1	169.7	20.52219	630	81.6	7.424932	12.0059	20.14477	4.168992
24185	1	21	56.5	171.3	19.25457	596.33	88.48	8.505529	8.0634	14.26509	2.747916
24299	1	47	102.8	170.2	35.48739	324.01	47.61	8.423307	26.3869	25.60527	9.10897
24389	1	30	95.5	179	29.80556	451.42	64.97	8.250399	24.7826	25.54112	7.734652
24466	1	32	112.1	190.1	31.01997	429.14	53.76	7.181301	30.6577	27.31084	8.483505
24494	1	46	82.5	186.9	23.61758	417.68	53.73	7.374219	13.1116	15.62514	3.753506
24542	1	43	74.3	169.7	25.80032	483.17	63.27	7.506556	21.2108	28.26482	7.36535
24631	1	38	115	186.4	33.09833	398.3	49.4	7.109835	34.6425	29.62384	9.970511
24641	1	24	98.7	169.9	34.19246	416.34	56.59	7.791739	28.931	28.95909	10.02251
24655	1	26	58.8	170.1	20.32211	581.1	67.03	6.612432	17.7057	29.64299	6.119338
24693	1	43	87.7	177.9	27.71071	483.45	56.88	6.744517	24.2993	27.59556	7.67789
24800	1	25	80.2	184.3	23.61151	391.22	51.51	7.547678	11.8264	14.63354	3.481785
24818	1	42	86.4	174.9	28.24452	459.76	67.15	8.372549	22.3131	25.60272	7.294244
24826	1	36	100.2	182	30.24997	401.81	53.04	7.567033	25.7748	25.62454	7.781307
24983	1	30	91	184.7	26.67519	435.22	62.95	8.291436	17.414	18.95009	5.104636
25034	1	29	76.6	184.1	22.60066	607.97	75.19	7.089585	17.7934	23.13132	5.249904
25105	1	36	71.5	183.4	21.25726	595.76	88.83	8.547344	13.4064	18.50059	3.985782
25109	1	25	97.4	185.9	28.18384	489.59	63.41	7.424515	25.4788	26.05519	7.372592
25110	1	22	124.2	195.4	32.52913	432.13	55.66	7.383659	33.286	26.45691	8.717912
25156	1	22	83.6	186.7	23.98378	461.22	54.02	6.714123	16.0062	18.86078	4.591976
25199	1	37	74.5	181.3	22.66526	497.69	71.54	8.240108	18.4314	24.45277	5.607415
25254	1	42	103.9	179.5	32.2468	481.84	69.86	8.311293	36.289	34.70247	11.26279
25265	1	23	70.1	175.1	22.86366	472.56	71.47	8.669812	11.9378	16.81238	3.893606

25345	1	26	70.4	159.4	27.70742	495.24	62.91	7.281935	24.0369	34.13014	9.460233
25367	1	32	72.3	174.8	23.66222	548.39	66.95	6.998483	20.7772	28.39754	6.799926
25374	1	41	81.9	173.7	27.14465	396.6	55.24	7.984428	19.028	22.94747	6.306574
25383	1	26	86.3	185.1	25.18825	593.63	72.57	7.007839	28.2633	32.87436	8.249166
25438	1	31	97.2	177.1	30.99054	408.11	58.88	8.270532	30.4739	31.11286	9.716076
25449	1	36	67	186.7	19.22145	494.63	58.28	6.754325	13.7406	20.18957	3.942004
25525	1	24	72.8	165.5	26.5788	485.79	62.32	7.353968	17.2439	23.4175	6.295634
25671	1	22	96.2	183.7	28.50734	436.26	52.64	6.916929	22.5318	23.23328	6.676942
25694	1	21	98.8	175.6	32.04114	444.12	60.68	7.832278	30.048	30.05846	9.744657
25756	1	22	58.7	177.3	18.67328	506.8	72.85	8.240163	8.8089	14.92192	2.802233
25790	1	28	125.4	187	35.86033	435.3	53.91	7.099431	43.3387	34.5298	12.39346
25816	1	42	73.6	179.2	22.91932	648.59	77.11	6.815274	14.9444	20.34334	4.653744
25944	1	46	105	178.7	32.88063	459.35	66.19	8.260218	37.0411	35.14933	11.59938
25963	1	22	62.3	179.3	19.37883	557.74	73.03	7.506066	11.7736	18.42507	3.662256
26003	1	34	104.8	180	32.34568	453.09	62.39	7.893568	33.5745	31.84497	10.3625
26004	1	21	75.4	175.9	24.36911	442.79	67.12	8.689544	14.5587	19.08699	4.705339
26027	1	45	79.5	171	27.18785	451.03	57.62	7.323365	19.92	24.66186	6.812353
26170	1	39	70.6	166.3	25.5282	467.67	65.89	8.076494	16.297	22.83497	5.89282
26250	1	36	78.9	183.9	23.32994	578.24	75	7.435257	21.2812	26.62803	6.292637
26368	1	31	106.4	171.8	36.04917	414.08	48.64	6.733675	36.7849	34.40272	12.46302
26432	1	48	68.3	176.6	21.89976	486.82	68.94	8.117938	14.3762	20.8026	4.609594
26545	1	31	77.5	174	25.59783	566.22	76.35	7.729772	20.9224	26.80994	6.910556
26566	1	36	95	177.5	30.15275	427.91	50.87	6.814785	26.3003	27.44074	8.347645
26609	1	43	63.8	162	24.31032	609.24	70.81	6.662681	21.5349	33.49822	8.205647
26681	1	21	42.8	163.5	16.01062	666.26	61.46	5.288003	8.3341	19.2999	3.11762
26750	1	43	96.5	179.4	29.98351	442.39	59.73	7.739806	25.5878	26.53876	7.950383
26841	1	25	102.9	174	33.98732	403.94	56.34	7.995449	29.8701	28.66513	9.865933
26854	1	47	84.3	171.2	28.76206	453.71	55.79	7.048892	22.0464	25.88101	7.521945
26876	1	42	73.2	172	24.7431	469.88	57.94	7.068616	15.6807	21.25776	5.300399
26952	1	30	98.9	176.4	31.78331	385.03	49.46	7.363807	28.3414	28.44939	9.108023
27059	1	48	65.1	170.5	22.39403	490.43	69.19	8.087404	12.8757	19.63436	4.429167
27111	1	35	74	176.9	23.647	596.16	63.5	6.105957	21.4036	28.80382	6.839607
27167	1	33	83.9	177.8	26.53985	434.82	63.12	8.321475	17.9197	21.08585	5.668488

27224	1	27	103.6	173.7	34.33682	396.36	54.36	7.86199	36.9731	35.42404	12.25424
27317	1	47	107.1	183.2	31.91086	416.29	46.99	6.470716	31.2003	28.81476	9.296251
27483	1	31	73.4	170.4	25.27882	584.77	72.63	7.119899	21.3977	28.75092	7.369328
27489	1	32	95.3	177.7	30.17992	396.1	56.58	8.188436	21.8862	22.82904	6.930994
27530	1	26	102.5	170.8	35.13566	366.11	51.78	8.107619	31.7613	30.73071	10.88736
27532	1	31	73.1	179	22.81452	439.22	57.2	7.465464	9.983	13.36284	3.115696
27632	1	21	90.6	174.1	29.89033	517.45	75.12	8.322045	30.102	32.74224	9.93111
27759	1	21	65.8	172.8	22.03629	567.82	73.24	7.394018	11.3908	17.24363	3.814756
27840	1	46	87.7	175	28.63673	446.8	57	7.313151	26.9036	30.34462	8.784849
27845	1	21	70.9	171.5	24.1056	518.26	77.36	8.556805	16.7049	23.431	5.679572
27929	1	34	132.4	182.2	39.88331	316.31	45.86	8.311205	43.5132	32.58532	13.10763
27932	1	41	89.4	181.7	27.07869	500.11	63.98	7.333673	26.7801	29.48097	8.111522
27979	1	47	84	167.9	29.79737	414.04	54.8	7.587193	27.8038	32.88841	9.862858
28000	1	46	87.5	174.8	28.63685	529	68.61	7.434891	26.201	29.67237	8.575017
28059	1	44	95.1	173.1	31.7385	406.36	49.89	7.037938	33.1555	33.80961	11.06526
28225	1	46	66.3	170.2	22.88729	441.92	67.94	8.81302	12.0585	18.09838	4.162691
28233	1	24	133.8	173.1	44.65417	421.34	52.26	7.110163	55.0382	41.13984	18.36835
28276	1	32	93.5	178.9	29.21399	443.6	66.61	8.607772	25.4033	27.0743	7.937239
28345	1	35	107.9	183.5	32.04419	386.3	52.99	7.863431	28.1975	25.842	8.374106
28365	1	41	69.2	175.5	22.46735	476.15	42.58	5.126308	12.2645	17.50211	3.981948
28376	1	33	66.2	175.5	21.49333	522.96	78.81	8.638846	11.8654	17.49963	3.852371
28399	1	27	66.2	175.5	21.49333	453.07	62.06	7.852163	9.5143	14.24277	3.089033
28423	1	45	77.5	182.3	23.31999	542.51	77.21	8.158469	20.5046	25.96834	6.169898
28569	1	35	88.1	166	31.97126	340.65	55.11	9.273953	21.7305	24.38476	7.885941
28571	1	27	79.9	179.3	24.85342	523.61	69.12	7.56726	23.2315	28.84962	7.226311
28605	1	33	71.6	184.6	21.01114	558.76	72.07	7.393874	9.5105	13.23955	2.790872
28658	1	47	99	174.4	32.54934	417.02	51.35	7.058728	26.9089	26.64807	8.847142
28722	1	21	71.1	177.4	22.5924	485.17	69.22	8.178629	11.8104	16.58634	3.752817
28749	1	39	71.6	168.1	25.33831	474.88	59.99	7.241655	18.4192	25.4845	6.518315
28756	1	24	66.4	176.4	21.33885	537.63	72.31	7.710059	11.9444	17.88453	3.83855
28838	1	40	80.8	172.1	27.28033	460.14	65	8.097785	22.2677	27.23621	7.518196
28861	1	26	126.9	183	37.89304	375.63	46.32	7.068889	42.4007	33.15093	12.66108
28954	1	47	82.1	172.9	27.46333	470.78	61.14	7.444753	26.7411	32.37982	8.945184

28961	1	31	75.9	174.8	24.84042	406.02	47.62	6.723336	15.3832	20.10169	5.034587
28992	1	37	112	192.8	30.13034	426.65	53.9	7.242023	27.2377	24.15229	7.327509
29067	1	32	63.4	166.3	22.92476	538.71	68.53	7.292368	13.9911	21.76909	5.059031
29070	1	49	93.1	182.5	27.95271	486.61	59.66	7.028216	26.2872	27.95803	7.892573
29373	1	37	108	175	35.26531	425.12	58.08	7.831734	40.0815	36.77809	13.08784
29374	1	35	66	180.4	20.28014	569	72.49	7.303124	9.4782	14.18166	2.912412
29441	1	24	72.7	177	23.20534	403.04	59.23	8.424351	9.3914	12.80545	2.99767
29592	1	48	81.4	183.7	24.1216	557.67	67.39	6.927253	16.9831	20.61892	5.032672
29751	1	31	78.7	185	22.99489	521.24	58.1	6.389712	19.6077	24.70759	5.729058
29846	1	41	106.1	177.3	33.75188	348.86	46.98	7.719776	28.9062	27.01724	9.195462
29854	1	49	76.8	181.2	23.39079	501.18	60.56	6.926837	17.9249	23.12953	5.459343
29889	1	23	90.5	179.9	27.96316	508.74	68.87	7.760274	24.9493	27.1378	7.708964
29908	1	27	87.7	169.8	30.41755	416.18	59.68	8.220353	22.4243	25.26166	7.777563
29994	1	34	98.7	186.1	28.49866	466.89	64.45	7.913183	29.5654	29.60225	8.53672
30047	1	24	79.8	186	23.06625	422.28	56.94	7.72965	13.2287	16.34295	3.823766
30084	1	47	60.3	175	19.6898	635.95	65.27	5.883469	13.5512	22.26458	4.424882
30201	1	26	108.3	166.7	38.97241	402.32	54.83	7.81249	43.4198	39.59767	15.62488
30244	1	45	79.3	186.1	22.8971	462.78	54.28	6.723697	16.1216	20.08239	4.654954
30303	1	38	79.9	177.2	25.44599	528.64	70.53	7.648156	21.1107	26.25817	6.723187
30319	1	24	114.3	189.3	31.89664	316.05	47.46	8.608249	22.6893	19.34645	6.331693
30345	1	25	92.5	177.9	29.22738	483.91	56.33	6.672952	32.2383	34.37106	10.18639
30382	1	48	80.9	177.5	25.67744	388.47	55.49	8.18842	16.3979	20.05393	5.20465
30428	1	21	112	184.5	32.90223	369.25	52.09	8.0868	22.8223	20.12298	6.704504
30451	1	44	120	172.7	40.23431	317.95	45.53	8.208838	39.8326	32.83414	13.35531
30498	1	27	115.7	182.4	34.77633	452.19	66.69	8.454397	38.299	32.4559	11.51166
30511	1	23	92.3	179.2	28.74258	520.21	69.6	7.669612	26.6984	28.6585	8.313985
30521	1	26	89.4	187.4	25.45648	571.42	66.32	6.653221	25.8439	28.84167	7.359001
30549	1	34	65.3	173.2	21.76794	537.77	75.39	8.036372	10.8594	16.50515	3.620012
30594	1	43	78.5	187.8	22.25761	459.37	63.42	7.91419	14.5131	18.24097	4.114992
30704	1	34	100	190.9	27.44026	443.54	63.36	8.188894	25.1627	24.97858	6.904709
30745	1	36	69.6	163.6	26.00415	419.44	62.98	8.607473	14.1336	20.10704	5.280636
30774	1	46	73.4	169.3	25.60838	468.25	58.66	7.181367	15.4872	20.81179	5.403299
30792	1	45	66.5	180.8	20.34346	543.98	60.73	6.399753	10.2929	15.38858	3.14877

30812	1	49	77.4	178.3	24.3466	516.67	60.15	6.673678	22.5008	28.7649	7.077751
30864	1	48	102.7	186.9	29.40031	428.48	54.97	7.354244	27.9807	26.84134	8.010138
31058	1	23	86.1	178.3	27.08323	470.47	63.44	7.729904	19.1095	22.09661	6.010999
21129	1	37	74.2	173	24.79201	458.25	59.11	7.394373	22.6946	30.16423	7.582813
21714	1	28	70.8	165.8	25.75516	443.55	63.84	8.250745	18.156	25.37069	6.60467
21781	1	36	105.7	174.1	34.87205	411.16	53.55	7.46606	34.7783	32.70076	11.47389
21912	1	22	61.3	174.6	20.10814	503.17	61.16	6.967799	11.3415	18.29501	3.720335
22042	1	29	68.1	171.1	23.262	587.67	76.22	7.434954	15.7698	23.02813	5.386742
22197	1	46	63	181.1	19.20895	536.03	59.65	6.37917	11.5031	18.0604	3.507341
22533	1	27	82.2	176.3	26.44644	531.58	63.2	6.815399	23.7848	28.53277	7.652351
23007	1	32	49.7	166.3	17.97099	632.45	72.28	6.55141	11.1439	22.28317	4.029514
23582	1	30	83.6	176.4	26.86638	420.67	51.35	6.997482	22.9836	27.10215	7.386197
23742	1	31	101.6	187.3	28.96131	423.98	56.87	7.689192	27.3202	26.70784	7.787684
24144	1	23	110.2	184.6	32.33838	359.32	51.52	8.219347	29.7626	26.85428	8.733885
24244	1	31	87	171.9	29.44201	473.8	54.9	6.642325	25.9599	29.51625	8.785192
25062	1	27	65.1	161.4	24.99044	443.6	59.74	7.719986	14.8141	22.54178	5.686803
25402	1	36	116.6	175.4	37.90001	389.43	49.27	7.252638	36.6575	31.31024	11.91526
25480	1	21	88.8	179.6	27.52963	430.55	55.46	7.384127	25.9711	28.88415	8.051516
25686	1	21	74	174.3	24.35774	471.78	54	6.561409	21.6812	29.07239	7.136554
25786	1	34	104.3	183.4	31.00885	451.87	61.18	7.761378	31.722	30.12802	9.43109
25991	1	39	80.9	177.1	25.79357	458.26	60.82	7.608119	20.9155	25.64136	6.668546
25998	1	25	63.7	179.3	19.81431	486.76	50.99	6.005	14.023	21.80847	4.361947
26024	1	23	70.8	192	19.20573	507.51	60.07	6.785094	9.1863	13.04524	2.491943
26285	1	40	71.6	165.8	26.04618	468.07	56.73	6.94776	20.8126	28.78282	7.57107
26580	1	25	113.8	183.1	33.9442	380.39	54.2	8.16795	29.7475	25.92605	8.873067
26624	1	31	75	171	25.64892	427.05	53.42	7.170807	20.2039	26.76676	6.909442
26692	1	30	78	177	24.89706	537.39	60.37	6.439831	26.344	33.65415	8.408822
27001	1	38	112.4	180	34.69136	366.47	55.95	8.751944	35.7564	31.53241	11.03593
27115	1	22	65.9	163.8	24.56165	583.3	82.5	8.107834	22.5632	34.00064	8.409553
27187	1	31	69.7	164.2	25.85154	466.88	60.64	7.44555	19.8422	27.94294	7.359419
27379	1	21	75.2	177.3	23.92216	389.66	57.68	8.485595	15.4711	20.43811	4.921571
27758	1	38	71.3	174.2	23.49596	479.83	56.62	6.764338	19.577	27.16884	6.451339
27833	1	21	63.4	162.5	24.00947	502.54	69.91	7.974648	18.426	28.79868	6.977893

27886	1	34	96	171.5	32.63946	430.79	47.71	6.348727	37.6739	38.9305	12.80891
27992	1	49	57.4	166	20.83031	543.57	64.14	6.764198	16.0424	27.72178	5.821745
28023	1	26	111.6	182.2	33.61766	399.35	50.95	7.313636	43.4771	38.76474	13.09676
28119	1	27	80.6	173.8	26.68305	434.67	58.23	7.679448	19.3361	23.62841	6.401317
28137	1	44	95	178	29.98359	448.97	64.22	8.19966	26.5718	27.8168	8.386504
28292	1	29	75.4	166.5	27.19837	400.28	59.46	8.515377	20.0636	26.34813	7.237363
28446	1	22	84.2	170.4	28.99832	458.76	62.92	7.862235	27.9365	32.92651	9.621279
28527	1	29	64.2	179.9	19.83685	528.31	65.24	7.078936	16.7049	25.65722	5.161567
28556	1	43	54	171.9	18.27435	504.7	64.21	7.293101	8.0354	14.7349	2.719291
28606	1	32	71.5	180.1	22.0434	500.84	78.61	8.997496	12.6104	17.35387	3.887778
28843	1	22	62.8	185.3	18.28979	564.41	62.51	6.348888	11.8529	18.86545	3.452024
29331	1	32	44.1	160.5	17.1194	609.89	54.54	5.126329	7.0149	15.8357	2.723149
29482	1	41	104.8	180.9	32.02463	385.28	46.83	6.967718	28.6455	27.07299	8.75345
29633	1	38	88.6	171.9	29.98348	397.01	54.17	7.821684	20.9667	23.50148	7.095424
29755	1	22	53.8	171.3	18.33444	556.04	59.91	6.17641	11.0181	20.41092	3.754845
29865	1	46	54.2	165.6	19.76417	531.45	58.02	6.258326	10.4661	19.28531	3.816491
30448	1	21	73.2	173	24.45788	457.45	56.57	7.089007	17.085	23.28358	5.70851
30514	1	39	77.2	171.8	26.15598	490.18	57.5	6.724424	25.6858	33.20196	8.702555
30665	1	23	84.9	174.1	28.00981	436.88	56.74	7.445091	24.7652	29.07516	8.170418
21017	2	37	45.1	152.7	19.34187	704.05	79.97	6.511281	16.0801	35.13923	6.896213
21048	2	42	65.4	173	21.85172	605.74	61.64	5.833366	23.3419	35.15971	7.799091
21115	2	32	93	147.8	42.57298	514.18	60.95	6.795187	47.8133	51.36758	21.88769
21116	2	38	90.4	154.6	37.82243	492.67	60.06	6.988308	38.8443	42.90453	16.25206
21122	2	37	52.9	153.8	22.36367	599.59	69.27	6.622678	18.6892	34.95432	7.900927
21178	2	45	81.6	162	31.09282	537.47	56.3	6.004779	36.3742	44.23261	13.86001
21279	2	40	86.3	161.3	33.16974	433.92	56.36	7.445677	34.33	39.82192	13.19487
21353	2	48	78.3	160.9	30.24473	513.24	59.11	6.602119	30.9684	39.06418	11.96208
21378	2	49	93.6	157.2	37.87658	441.25	50.2	6.521716	44.5219	47.14894	18.01642
21409	2	45	71.4	153.9	30.14539	471.06	51.84	6.30858	30.499	42.44154	12.87681
21426	2	37	71.9	161.1	27.7037	589.57	69.99	6.80524	30.7128	42.37382	11.83391
21849	2	45	73.4	157.6	29.55178	517.47	60.79	6.734259	26.954	36.20339	10.85203
22000	2	34	55.5	151	24.34104	659.08	74.28	6.460656	22.6401	40.53733	9.929433
22094	2	32	75.3	169	26.36462	541.38	62.45	6.612613	26.6174	34.95244	9.319492

22261	2	42	67.3	156.7	27.40798	540.9	71.79	7.608339	26.1063	38.86717	10.63181
22308	2	41	67.7	154.2	28.47213	518.66	68.38	7.557692	27.4036	40.15997	11.52495
22449	2	28	84	158.2	33.56343	536.42	62.83	6.714365	36.6762	43.22093	14.65451
22516	2	40	86	150.1	38.17131	503.79	62.93	7.160627	39.1001	45.28745	17.35467
22549	2	31	53.4	162.5	20.22249	697.7	69.89	5.742344	17.15	31.70565	6.494675
22730	2	49	57.9	144.6	27.69121	656.7	83.54	7.292397	24.3465	41.7293	11.64394
22786	2	38	68.7	165.1	25.2036	500.07	58.66	6.724409	23.1195	33.26154	8.481727
22798	2	47	69.4	157.4	28.01238	574.11	67.04	6.693939	30.0269	42.72741	12.11996
22858	2	31	78.2	153	33.40595	482.1	71.79	8.5363	32.8909	41.26134	14.05054
22873	2	24	70.4	159	27.847	581.74	62.79	6.187346	31.8372	44.59491	12.59333
22885	2	24	96.9	149	43.64668	448.39	51.72	6.612192	49.8699	51.83216	22.46291
22900	2	34	71.6	181.3	21.78299	579.97	69.47	6.866487	22.7495	31.44589	6.921118
23127	2	40	48.3	155.2	20.05228	795	74.45	5.368345	16.9263	34.8709	7.02714
23205	2	22	72.8	161.3	27.98096	576.92	72.47	7.200879	30.9118	42.176	11.88107
23315	2	28	62.8	161.2	24.16738	630.84	67.53	6.136495	24.8362	39.16165	9.557737
23384	2	39	75.7	158.8	30.01891	525.68	59.15	6.450244	29.6532	39.12119	11.759
23450	2	23	68.6	170.7	23.54274	606.42	66.52	6.288131	25.7337	37.22384	8.831515
23466	2	48	56.3	158	22.55248	609.19	64.78	6.095805	21.2318	37.13704	8.504967
23467	2	48	82.4	157.8	33.09125	548.43	54.64	5.711265	41.5127	50.03164	16.6712
23481	2	49	78.2	161.6	29.94498	525.54	53.57	5.843307	35.6842	45.60223	13.66448
23583	2	34	94.1	159.2	37.12817	482.73	54.83	6.511137	45.1293	47.62233	17.80625
23659	2	23	92.5	159.1	36.54276	449.18	54.36	6.937482	44.5017	47.63045	17.5807
23748	2	37	52.1	144.1	25.09053	664.93	78.82	6.795217	23.689	44.89036	11.40824
23801	2	41	56	161	21.6041	529.27	56.84	6.156298	19.5522	34.49869	7.542996
23834	2	49	56.7	153.5	24.06392	545.53	54.16	5.691187	22.7436	39.72286	9.652559
23850	2	43	68.6	156.1	28.15259	514.36	59.33	6.612261	28.5157	41.15069	11.70249
24071	2	34	101.7	154.8	42.44036	400.59	50.82	7.272394	48.0961	47.02	20.07095
24095	2	30	81.9	156.6	33.39646	502.1	58.72	6.704072	36.8514	44.56982	15.02694
24109	2	30	75.5	161.5	28.94689	487.01	61.09	7.190765	26.8454	34.86081	10.29259
24114	2	41	67.6	150.3	29.92463	543.19	64.48	6.804812	27.4374	40.10014	12.14577
24222	2	43	101.2	156.9	41.10878	462.99	53.57	6.632739	47.5052	46.96603	19.29724
24270	2	25	80.7	166.5	29.11019	611.8	68.73	6.439909	35.0842	43.34802	12.65561
24309	2	38	89.9	163.9	33.46584	498.26	60.83	6.998495	41.5804	45.89919	15.47857

24393	2	41	66.1	148.5	29.97427	467.57	59.07	7.242078	26.7182	40.04208	12.11586
24471	2	22	56.3	159.6	22.10256	569.33	66.68	6.713892	18.9085	33.21978	7.423202
24482	2	33	88.5	163.6	33.06562	541.22	63.48	6.723663	39.3247	44.19558	14.69261
24489	2	41	66.5	155.2	27.60821	564.16	75.78	7.700079	27.3214	40.50801	11.34278
24568	2	33	62.8	151.1	27.50621	591.88	64.51	6.247931	26.5976	41.66989	11.64967
24582	2	38	49.6	163	18.66837	722.42	64.98	5.156236	15.6843	31.11835	5.903233
24676	2	37	114.3	163.7	42.65293	396.96	52.4	7.567064	53.4148	46.07866	19.93261
24783	2	21	62	158.4	24.71049	607.6	73.53	6.937287	23.8577	38.01673	9.508636
24881	2	32	66.5	144.7	31.7603	509.01	64.66	7.282026	25.0979	37.28337	11.98672
24982	2	29	72.8	170.1	25.1607	642.11	70.32	6.27787	29.939	40.38825	10.34734
25009	2	24	54.7	163.1	20.56267	682.52	68.12	5.721397	17.0665	30.66812	6.415589
25028	2	28	53.3	157.4	21.51383	652.64	65.6	5.761997	18.87	34.84089	7.616623
25107	2	48	81.4	155.6	33.62058	455.58	52.15	6.561944	33.9476	41.33333	14.02135
25317	2	21	48	149.8	21.39034	520.92	72.84	8.015706	12.322	25.4916	5.491078
25375	2	37	50.6	159	20.01503	628.24	88.52	8.07716	18.4062	36.01806	7.280646
25378	2	27	104.3	161	40.23765	449.73	51.88	6.612885	50.6433	48.13613	19.53756
25551	2	32	58.3	154.4	24.45535	652.29	65.22	5.731693	21.8095	36.87494	9.148526
25622	2	29	81.3	154.6	34.01509	483.37	54.05	6.410012	34.5045	42.47058	14.43633
25624	2	26	71.3	161.2	27.43844	565.69	61.85	6.26764	27.6526	38.32675	10.64157
25796	2	45	111.1	157.7	44.67357	430.43	46.76	6.227516	57.0083	50.85545	22.92317
25818	2	47	71.8	159.6	28.18764	605.58	65.25	6.176634	32.5539	45.03829	12.78019
25894	2	22	50.4	153.5	21.39015	655.78	78.78	6.886534	17.214	33.57526	7.305754
26007	2	24	44.6	159.2	17.59741	671.96	83.46	7.119964	12.8015	28.52244	5.050969
26035	2	44	69.9	156.8	28.43054	428.96	54.34	7.261824	20.7471	29.42884	8.438501
26064	2	30	79.2	168.4	27.92808	537.95	56.64	6.035652	30.3017	37.87196	10.6852
26070	2	21	48.3	148.6	21.87306	704.77	83.29	6.774673	16.0987	33.22581	7.290431
26075	2	31	70.2	154.7	29.333	534.24	66.07	7.089421	29.9832	41.99475	12.52845
26153	2	22	56.3	159.6	22.10256	689.03	75.22	6.258036	19.1746	33.64821	7.527669
26249	2	45	80.3	158.9	31.80298	564.35	67.79	6.885888	38.1158	47.21884	15.09584
26569	2	33	73.4	156.7	29.89221	543.27	65.65	6.927266	31.9136	43.01998	12.99684
26571	2	21	41.4	144	19.96528	643.66	74.7	6.652838	11.3168	26.98267	5.457562
26645	2	30	94.5	157.8	37.95053	470.28	56.33	6.866353	43.2031	45.81326	17.35006
26752	2	37	56.7	160	22.14844	685.8	76.68	6.409549	21.0234	36.70382	8.212266

26753	2	41	96.4	157.1	39.05932	538.71	57.29	6.096304	47.2718	48.52321	19.15357
26786	2	37	56.7	165.6	20.6758	649.82	76.34	6.734447	18.721	32.7584	6.826662
26864	2	41	80.7	146.6	37.54962	453.8	58.94	7.445408	37.9781	45.9937	17.67117
26986	2	42	79.7	160.1	31.09393	544.65	76.74	8.076945	28.8981	35.97486	11.27422
26998	2	31	63.3	155.6	26.14475	586.1	61.6	6.024928	26.552	41.14988	10.96675
27040	2	37	48.1	156.3	19.68916	747	79.7	6.116184	17.0922	35.08583	6.996487
27358	2	41	60.7	159.4	23.88977	569.97	65.24	6.561525	21.2889	34.84825	8.378699
27398	2	23	95.8	159.3	37.75148	416.71	49.69	6.83562	38.3819	39.54614	15.12498
27506	2	22	82.8	167.5	29.51214	571.06	60.83	6.106311	35.6805	42.71037	12.71749
27611	2	42	63.2	162.6	23.9043	573.51	58.36	5.833338	24.82	38.82001	9.387732
27708	2	42	66.3	153.3	28.2117	541.66	71.89	7.608247	22.9641	34.44784	9.771587
27761	2	45	67.5	158.6	26.83474	621.77	67.77	6.248138	28.0685	41.14029	11.15868
27854	2	46	122.6	167.9	43.48997	373.63	46.21	7.089851	58.3475	47.10116	20.69764
27882	2	49	72.2	152.9	30.8832	498.71	54.97	6.318595	32.2881	44.31757	13.81108
27935	2	21	59.8	168.6	21.03711	623.49	62.56	5.751884	20.8314	34.70795	7.328301
28145	2	30	80.9	161.4	31.05571	624	60.08	5.519353	38.1432	46.64774	14.64233
28151	2	43	84	160.9	32.44645	491.54	61.22	7.139656	30.6378	36.36146	11.83438
28161	2	32	57.4	158.5	22.84827	548.77	61.84	6.459843	22.0492	38.06719	8.776762
28266	2	41	70	161.5	26.83818	592.3	67.59	6.541594	30.4985	42.94172	11.6932
28315	2	25	92.4	169.7	32.08546	549.89	58.67	6.11622	39.4611	42.29816	13.70268
28315	2	25	92.4	169.7	32.08546	549.89	58.67	6.11622	40.5325	43.76026	14.07472
28315	2	25	92.4	169.7	32.08546	549.89	58.67	6.11622	39.713	42.34342	13.79015
28315	2	25	92.4	169.7	32.08546	549.89	58.67	6.11622	40.3529	43.22489	14.01235
28315	2	25	92.4	169.7	32.08546	549.89	58.67	6.11622	38.9403	41.6876	13.52183
28434	2	38	85.6	156.8	34.81622	509.84	63.5	7.139745	39.6602	45.94018	16.13106
28592	2	41	90.9	169.2	31.75142	474.04	43.14	5.216846	42.1631	45.928	14.72759
28639	2	44	69.1	161.6	26.46033	580.19	61.9	6.115941	28.0961	40.36065	10.75879
28808	2	24	96.3	156.2	39.46974	451.66	54.26	6.886698	45.7093	46.72663	18.73452
28822	2	26	83	160.2	32.34097	598.9	59.36	5.681754	38.1081	45.3122	14.84883
28837	2	40	50.3	156.1	20.64249	682.27	70.27	5.904138	17.1867	33.56404	7.053208
28985	2	32	97.4	163.4	36.48	474.26	52.78	6.379634	42.9702	43.5756	16.09397
29054	2	44	60	150.3	26.56032	554.85	67.83	7.007919	24.7646	40.95475	10.96259
29056	2	25	78.9	155.8	32.5044	545.17	62.4	6.561385	37.7359	47.26594	15.54604

29122	2	41	69.2	163.2	25.98159	603.15	63.07	5.994326	27.0507	38.49286	10.15636
29125	2	46	85.3	153.1	36.39139	421.78	58.53	7.954912	36.3103	42.27806	15.491
29155	2	40	63.4	168.4	22.35657	679.68	60.42	5.095879	25.5943	39.93674	9.025247
29212	2	47	80.4	152.2	34.70777	459.56	55.04	6.865609	32.7668	40.01035	14.14506
29251	2	38	67.9	145.6	32.02927	521.83	69.9	7.678758	27.5651	40.22282	13.0028
29682	2	46	46.8	148.6	21.19377	537.56	58.87	6.277836	14.1694	29.87944	6.416731
29848	2	28	68.9	164.4	25.49269	667.02	76.12	6.541883	27.6286	39.74812	10.22246
29966	2	43	79.6	161.7	30.44339	639.18	64.14	5.752394	39.0578	48.21266	14.93784
29972	2	21	101.3	169	35.46795	471.33	56.45	6.865651	46.7131	45.83486	16.35555
29974	2	24	75.9	159.7	29.75993	587.56	72.56	7.079261	34.3618	45.07772	13.47305
30112	2	28	126.7	167.4	45.21325	442.89	46.32	5.995364	60.3992	47.97806	21.55362
30279	2	37	87.7	150.7	38.61652	575.76	68.66	6.836049	45.9317	51.61445	20.22488
30331	2	47	60.8	156.4	24.85593	576.69	62.14	6.176916	25.3417	41.35327	10.36006
30386	2	35	48	153.5	20.37157	753.44	87.58	6.66345	18.2758	37.60971	7.75639
30426	2	36	59.6	153.3	25.36074	612.48	71.08	6.652707	22.5158	37.35878	9.580829
30450	2	42	62.3	156.5	25.43662	561.86	73.57	7.50612	23.0919	36.63834	9.428248
30522	2	30	83.6	161.1	32.21182	613.13	64.98	6.075332	40.6613	48.12763	15.66716
30634	2	25	61.2	165.3	22.39782	625.55	58.8	5.388379	20.0616	32.48495	7.342093
30669	2	25	100.5	169.6	34.93931	523.3	59.07	6.470817	46.2897	45.59362	16.09284
30695	2	28	58.9	152.3	25.39309	629.65	68.07	6.197255	23.689	39.81686	10.21285
30840	2	24	70.3	159.2	27.73762	462.55	54.34	6.734476	27.1376	38.37824	10.70743
30855	2	49	100	160.9	38.62673	471.5	50.22	6.105734	47.624	47.05682	18.39559
30895	2	22	54	152.8	23.12848	559.97	74.52	7.628707	18.1194	33.30625	7.760632
30935	2	41	55.4	150.5	24.45889	551.36	64.57	6.713336	21.8331	39.00474	9.639231
30977	2	45	72.1	154.2	30.32261	499.29	69.02	7.924374	31.5968	43.04512	13.28845
30999	2	23	81.2	155.4	33.62436	588.24	58.4	5.691165	39.4252	48.15303	16.3257
31092	2	36	61.1	150	27.15556	560.05	64.4	6.591768	24.0265	39.02433	10.67844
21220	2	44	61.4	165.1	22.52549	649.03	62.38	5.509643	23.22	37.49443	8.518597
21517	2	42	101.9	161.9	38.8759	397.41	48.44	6.987281	43.9632	43.01919	16.77241
21726	2	41	60.5	153.5	25.67667	564.48	57.64	5.853536	23.0457	37.82448	9.780772
21854	2	40	72.3	160	28.24219	618.71	64.37	5.964022	32.0777	43.99056	12.53035
21861	2	23	67.8	154.5	28.40356	576.81	71.44	7.099888	29.1408	42.21023	12.208
22575	2	43	70.6	155.3	29.27263	527.98	68.39	7.425368	28.3872	39.81481	11.77009

23373	2	37	66.4	167.5	23.66674	644.97	64.15	5.701643	23.4412	35.05593	8.355072
23660	2	29	56.2	159.4	22.1187	564.93	73.97	7.505918	18.9517	33.21183	7.458844
24430	2	39	67.7	153.4	28.76987	564.98	75.08	7.617879	30.9562	45.31066	13.15518
24630	2	23	66.7	145.8	31.37695	518.69	63.78	7.04887	28.2259	42.10615	13.278
24733	2	48	74.8	157.2	30.26889	494.8	46.08	5.338579	31.552	41.88287	12.76797
25029	2	40	52.8	153.7	22.35045	647.08	72.93	6.460871	19.6839	36.91633	8.332272
25611	2	24	59.8	163.1	22.47984	629.57	65.17	5.933986	21.2679	35.39912	7.994967
25766	2	24	56.3	170.3	19.41239	589.18	64.63	6.288239	13.0311	22.75392	4.493159
26230	2	36	90.9	154.2	38.22919	410.18	52.98	7.404237	37.0815	40.38856	15.59511
26461	2	23	85.7	154.9	35.71725	461.73	62.84	7.80173	36.1976	42.14708	15.0861
27200	2	25	57.6	159.3	22.69817	562.72	71.19	7.252195	17.8732	30.89411	7.043212
27216	2	27	66	156.5	26.9473	544.69	64.76	6.81554	24.6759	36.63537	10.07498
27300	2	47	59.6	156.6	24.30316	634.22	59.17	5.348161	25.8012	42.84142	10.52098
28176	2	25	50.8	159.6	19.94334	690.78	75.53	6.267908	16.9799	33.14768	6.666062
28421	2	38	79.3	157.5	31.96775	465.26	57.04	7.027918	32.4642	40.72191	13.08711
28469	2	49	95.1	162	36.23685	456.33	51.83	6.51096	41.64	43.81474	15.86648
29466	2	47	90.8	157.9	36.41846	413.74	51.97	7.20059	37.1858	40.79403	14.91464
29859	2	29	63.6	165.4	23.24803	649.71	62.79	5.540051	24.1189	37.41157	8.816303
30143	2	43	82.5	156	33.90039	489.3	56.01	6.561954	35.7675	43.02779	14.69736
30403	2	35	94.9	159.1	37.4909	450.06	40.96	5.217139	41.422	43.16766	16.36405
30409	2	23	45.9	149.7	20.48185	635.38	72.17	6.511275	16.1205	34.77333	7.193412
30477	2	44	104.8	160.5	40.68283	509.81	55.47	6.237243	56.1418	53.35623	21.79397
30585	2	35	81.4	150.7	35.84247	538.42	59.82	6.368954	39.143	47.88574	17.23565
30612	2	40	69.1	163.7	25.7858	560.66	60.91	6.22776	26.4672	37.84516	9.876672
21031	2	37	52.3	160.8	20.22691	628.89	64.66	5.893915	16.0425	30.50027	6.204402
21052	2	37	54.4	164.9	20.00588	697.29	73.9	6.075386	21.0119	38.26837	7.727233
21091	2	25	57.4	161.2	22.08929	656.4	72.93	6.369136	21.0072	36.38322	8.084219
21142	2	31	95.2	154.8	39.72785	465.04	58.09	7.160674	46.9728	49.13791	19.60219
21147	2	31	91.9	165.4	33.59267	505.01	57.9	6.572361	39.7759	43.04164	14.53948
21184	2	47	95.5	161.7	36.52442	495.14	57.29	6.632751	44.19	46.19515	16.90067
21223	2	34	55.7	153.9	23.51679	539.52	52.71	5.60052	21.5396	38.23199	9.094114
21297	2	46	90.1	170	31.17647	539.07	53.9	5.73174	40.5264	44.73132	14.02298
21303	2	34	80	168.5	28.1767	513.82	63.18	7.04874	31.4356	38.93624	11.07189

21313	2	22	85.4	156.6	34.82366	562.6	61.61	6.27761	42.0065	48.62843	17.12904
21351	2	40	63.2	172.9	21.14108	780.04	84.6	6.217222	25.2087	39.65615	8.43258
21356	2	36	72.2	166.2	26.13817	565.94	73.5	7.444916	28.2633	38.8381	10.23201
21360	2	44	75.8	169.5	26.38334	609.61	68.06	6.40004	31.836	41.43737	11.081
21431	2	24	78	160.8	30.16633	563.02	62.36	6.34929	34.8219	44.43893	13.4673
21446	2	23	56.1	164.9	20.63106	686.89	68.32	5.701689	18.9199	33.03724	6.957889
21476	2	47	77	170.6	26.45652	511	54.16	6.07576	29.7912	38.13537	10.23599
21486	2	30	60.1	160.9	23.21466	503.01	60.07	6.845795	19.0611	31.32617	7.362679
21490	2	25	63.1	170.3	21.75705	542.94	65.41	6.906137	15.5999	24.50395	5.378888
21509	2	42	52.1	168.2	18.41559	654.44	59.67	5.226718	14.6524	27.73101	5.17913
21522	2	47	87.3	169.8	30.27881	434.69	42.77	5.640303	34.9008	39.42805	12.10487
21533	2	40	102.3	173.1	34.14141	524.2	51.31	5.611098	46.6394	44.80379	15.56535
21551	2	38	48.6	163.7	18.13589	651.14	67.86	5.974235	15.371	31.32464	5.735942
21568	2	43	59	162.1	22.4536	590.8	62.2	6.035215	20.6533	34.56446	7.860016
21660	2	41	57.5	152.4	24.75699	590.11	71.41	6.936956	22.0976	37.82611	9.514263
21685	2	49	77.7	163.1	29.20876	521.41	53.05	5.832421	33.8312	43.3107	12.71773
21719	2	35	58.9	166	21.37466	612.47	66.1	6.186706	20.0527	33.836	7.277072
21812	2	35	62.1	165.6	22.64493	563.8	61.95	6.298819	21.1862	33.66271	7.725603
21832	2	48	67.6	164.4	25.01169	601.78	54.87	5.22685	26.6173	39.33735	9.84828
21862	2	33	105.1	163.2	39.46048	516.52	47.01	5.217302	55.2926	51.9	20.75997
21869	2	36	122	171.8	41.33458	470.78	46.74	5.691327	61.5797	49.92298	20.8637
21871	2	44	75.6	168.6	26.59541	506.23	53.92	6.105832	28.046	36.7814	9.866333
21926	2	36	67.3	164.2	24.96139	551.78	57.41	5.964368	25.6131	37.68583	9.49983
22002	2	22	54.1	161.1	20.84521	638.22	64.49	5.792484	17.5767	32.1603	6.772457
22025	2	30	57.9	163	21.79231	621.27	71	6.551199	19.6841	33.64901	7.408672
22055	2	32	54.6	154.3	22.933	615.01	73.77	6.876073	21.4448	38.84407	9.007207
22059	2	25	56.4	157.7	22.67857	650.23	80.41	7.089015	18.4035	32.44154	7.400091
22068	2	33	115.7	175.7	37.47916	551.19	55.99	5.82307	53.9892	46.31757	17.48894
22115	2	40	60.6	162.4	22.97738	560.52	62.57	6.399085	23.8655	39.15503	9.048956
22206	2	25	60.7	159.5	23.85983	616.85	67.99	6.318418	24.2018	39.48459	9.513193
22216	2	49	79.9	159.3	31.48584	562.59	57.05	5.813083	33.5618	41.48236	13.22555
22280	2	31	56.4	147.6	25.88847	613.18	77.9	7.282699	20.7762	36.55079	9.536596
22299	2	29	64.3	165.4	23.5039	619.86	65.04	6.014919	23.8545	36.82989	8.719655

22378	2	30	50.8	158.2	20.29788	607.04	63.91	6.035238	15.1567	29.49187	6.056081
22404	2	42	80.2	165.8	29.17463	550.44	58.53	6.095529	33.4125	41.34709	12.15458
22443	2	38	57.8	158.2	23.09484	577.24	61.38	6.095556	19.5868	33.49081	7.826193
22545	2	24	105.2	172.8	35.23127	543.94	62.07	6.541444	49.0505	46.50959	16.42691
22555	2	36	54.4	161.3	20.90885	576.55	70.38	6.997697	17.1098	31.17436	6.576218
22574	2	47	113.3	161.1	43.65549	405.88	41.44	5.852817	54.2537	47.77347	20.90443
22586	2	32	53.2	164.8	19.58832	719.57	72.33	5.762199	18.6904	34.96781	6.881834
22619	2	43	45.6	155.7	18.80995	528.09	52.43	5.691343	13.2082	28.59202	5.448368
22620	2	26	57.7	171.3	19.66351	623.43	58.27	5.357969	17.1372	29.3509	5.840165
22665	2	23	61.8	167.1	22.13276	503.06	59.19	6.744836	19.6212	31.45598	7.027044
22689	2	28	121.2	166.6	43.66693	519.39	48.73	5.378308	62.6908	51.72043	22.58675
22757	2	33	80.2	157.5	32.33056	607.94	62.93	5.933895	37.0389	45.66891	14.93128
22758	2	35	65.7	172.6	22.05382	575.1	51.63	5.146377	23.3386	35.07834	7.834174
22808	2	39	54	162	20.57613	546.46	68.45	7.180554	14.9344	27.30782	5.690596
22809	2	42	82.2	168.6	28.91723	557.99	63.67	6.541108	36.6431	44.09588	12.89072
22818	2	40	52	150.4	22.98834	578.56	67.25	6.66326	18.3488	34.91558	8.111702
22825	2	39	60.2	158.7	23.90246	573.42	57.74	5.772272	23.7659	39.0864	9.436271
22878	2	45	104.8	166.1	37.98586	485.66	57.31	6.764581	54.1268	51.21604	19.61883
22896	2	34	115.3	168	40.85176	528.29	48.45	5.257318	60.0642	52.01336	21.28125
22915	2	22	58.5	162.8	22.07227	542.58	63.64	6.723714	15.6061	26.41878	5.888241
22918	2	35	122.8	170.5	42.2425	414.38	42.16	5.832365	60.5573	49.3142	20.83137
22959	2	31	66.3	166.3	23.97337	606.54	68.25	6.450391	25.4359	37.87313	9.197348
22999	2	33	71.4	167.2	25.54028	557.93	58.64	6.025001	26.0783	36.19201	9.328391
23048	2	43	54.3	154.8	22.6599	559.32	55.14	5.651312	16.2699	30.0441	6.789581
23059	2	39	82	167.1	29.36709	501.42	59.7	6.825202	31.8773	38.40898	11.41639
23218	2	28	44.3	153.3	18.85035	622.9	68.66	6.318709	10.0714	22.44518	4.28554
23271	2	34	71	168.5	25.00682	655.52	67.16	5.873103	29.0593	40.32799	10.23494
23318	2	28	46.6	158.1	18.64327	666.82	78.81	6.775098	14.9546	31.7809	5.982891
23323	2	21	59.4	170.1	20.52947	621.75	60.85	5.61032	21.2389	35.26803	7.340462
23381	2	49	90.8	160.1	35.42446	495.64	62.26	7.200881	42.818	47.02851	16.70489
23418	2	21	67.8	166.9	24.3398	446.84	77.83	9.984765	22.0904	31.91286	7.930324
23424	2	27	80.2	160.4	31.17207	545.6	56.67	5.954177	35.2375	43.78656	13.69608
23449	2	37	66.4	176.3	21.36306	531.72	54.67	5.893984	20.6724	30.85548	6.65099

23451	2	47	103.2	175.6	33.46807	456.64	45.74	5.742025	44.6994	43.00025	14.49615
23463	2	24	51.5	160.3	20.04196	661.47	71.39	6.186857	18.0584	34.77953	7.027684
23464	2	48	112.4	168.2	39.72961	498.34	49.3	5.671057	56.1886	49.78165	19.86078
23508	2	25	47.7	161.7	18.24309	596.14	72.14	6.936985	11.6991	24.23825	4.474375
23530	2	38	68.2	173.3	22.70844	509.39	61.73	6.946863	21.0933	30.69014	7.0234
23554	2	27	45.1	163.9	16.78876	691.96	70.53	5.842998	13.5498	29.60184	5.043999
23563	2	40	111.2	172.1	37.54422	527.24	52.9	5.75162	56.8736	50.71299	19.20211
23569	2	41	55.3	164.6	20.41106	622.32	68.93	6.349469	16.2318	28.93188	5.991106
23574	2	34	65.9	159.9	25.7744	687.45	66.31	5.529435	27.84	41.92342	10.88861
23586	2	41	73.3	158.4	29.21417	524.26	58.43	6.388987	31.3738	42.20227	12.50422
23592	2	22	49.6	169.9	17.18284	682.35	76.78	6.450357	12.6309	25.36422	4.3757
23602	2	43	85	165.2	31.14575	532.02	64.48	6.947682	31.4169	36.87352	11.5118
23607	2	43	64.8	174	21.40309	481.67	55.82	6.643288	16.2205	24.86163	5.357544
23610	2	40	85.9	163.2	32.25172	541.19	64.72	6.855381	35.7745	41.51735	13.43177
23637	2	40	60	161.2	23.08985	585.34	53.89	5.277677	21.0044	34.59582	8.083142
23647	2	37	70.4	160.8	27.22705	621.07	62.43	5.762297	29.0904	40.94114	11.25065
23724	2	34	84.2	165.9	30.59281	543.15	52.78	5.570478	38.8459	45.59431	14.11407
23819	2	36	81.8	160.9	31.59666	618.16	70.87	6.572103	38.6302	46.83964	14.92158
23899	2	24	76.8	169.4	26.76298	562.92	67.72	6.896252	30.7952	39.74923	10.7314
23912	2	25	56	161	21.6041	634.11	75.73	6.846147	17.6483	31.34216	6.808495
23919	2	26	61.8	172.2	20.84117	685.03	65.72	5.499596	20.4083	32.80534	6.88241
23950	2	33	54.5	153	23.28164	585.05	70.18	6.876433	19.8018	35.90424	8.459054
23961	2	39	62	159.9	24.24905	603.61	76.47	7.262356	22.2634	35.66864	8.707522
23964	2	45	73.7	175.7	23.87393	533.52	46.21	4.965101	26.9452	36.13856	8.728465
23993	2	27	80.7	170.7	27.69533	504.99	63.26	7.181072	33.1344	40.68521	11.37135
24050	2	35	95.3	166.9	34.21214	478.02	60.3	7.231262	38.9368	40.5443	13.97808
24084	2	43	53.3	156.1	21.87366	615.5	72.52	6.754179	16.8922	31.27826	6.932349
24086	2	40	79.4	162.8	29.95792	459.87	51.66	6.439649	33.4535	41.72113	12.62213
24118	2	32	82.6	162.4	31.31901	490.7	49.58	5.792064	34.774	41.63853	13.18507
24131	2	43	59.2	159.2	23.358	538.36	57.25	6.096008	21.5725	36.13962	8.511662
24195	2	46	90.1	178.9	28.15167	552.6	54.48	5.65157	37.5347	41.36652	11.72768
24204	2	39	67.5	170.8	23.13812	574.17	52.05	5.196646	24.8487	36.27961	8.51781
24279	2	46	69.8	157.8	28.03118	565.91	60.28	6.106168	32.4431	45.90567	13.02892

24282	2	36	69.1	157.1	27.99791	616.4	71.65	6.663408	30.9176	44.47545	12.52718
24305	2	34	56.7	162	21.60494	595.2	71.82	6.91712	18.0782	31.50802	6.888508
24359	2	45	67.6	165.7	24.62077	586.12	64.6	6.318134	22.0288	32.37853	8.023167
24392	2	25	54.1	164.7	19.9439	747.78	76.09	5.833062	19.0292	35.28899	7.015091
24418	2	47	61.7	164.7	22.74563	558.05	57.18	5.873729	19.6094	31.63899	7.228981
24445	2	29	55	157.7	22.11563	615.09	73.89	6.886362	21.2501	38.22215	8.544715
24452	2	36	102.4	164.8	37.70384	535.57	64.24	6.875941	49.834	48.57678	18.34895
24460	2	46	72.1	163	27.13689	555.7	60.27	6.217326	27.5153	38.01648	10.35617
24479	2	26	108.7	161.8	41.52145	464.45	56.7	6.99821	52.4791	48.1735	20.04607
24521	2	30	94.3	175.5	30.61663	590.02	60.24	5.852765	44.2746	46.35346	14.37475
24525	2	46	60.1	158.2	24.01383	553.41	61.68	6.389108	16.8779	28.05549	6.743812
24572	2	31	58.4	162.4	22.14322	647.27	62.32	5.51931	21.3613	35.26358	8.099452
24701	2	33	119.3	163.8	44.46442	427.91	52.69	7.058601	61.6428	51.09657	22.97495
24722	2	23	64.1	165.9	23.28977	587.91	64.07	6.247219	20.5224	31.70604	7.456506
24803	2	35	60	159.7	23.52564	486.61	61.73	7.272071	18.3551	30.03651	7.196924
24805	2	39	85.2	162.6	32.22541	557.73	56.36	5.792817	42.6125	49.7469	16.11744
24821	2	39	91	162.8	34.33465	540.62	60.45	6.409838	43.0629	46.84849	16.2478
24883	2	32	56.5	167.8	20.06617	539.34	68.42	7.272158	12.95	22.66347	4.599238
24908	2	34	56.6	154.1	23.83478	540.32	67.78	7.191068	18.55	32.52263	7.811576
24912	2	34	78.6	169.7	27.29348	514.73	58.37	6.500594	29.1552	36.62507	10.124
24955	2	42	73.3	161.7	28.03393	508.44	53.8	6.065763	28.963	39.08083	11.07703
24962	2	28	44.7	164.4	16.5388	789.29	82.68	6.004913	12.7306	28.47964	4.710264
24966	2	47	82.3	159.1	32.51318	529.69	53.06	5.742332	35.4379	42.63319	13.99999
24977	2	40	53	166.4	19.1412	556.96	58.54	6.025201	15.5779	29.15615	5.626033
24995	2	28	57.2	159.4	22.51228	642.26	74.08	6.612002	21.555	37.37405	8.483428
25013	2	42	73.6	171.3	25.08205	677.48	66.19	5.600654	30.8775	41.37034	10.52271
25041	2	24	59.6	160.1	23.25218	600	63.91	6.106051	21.9945	36.68587	8.580872
25045	2	40	80.3	162.8	30.2975	558.57	57.82	5.933943	35.3301	43.92166	13.33018
25090	2	22	120.1	164.1	44.59907	424.75	44.79	6.04492	58.9418	48.90831	21.88801
25122	2	22	64.7	162.2	24.59249	546.83	64.63	6.77524	24.065	36.91953	9.147116
25244	2	48	96.8	158.2	38.67786	424.04	41.2	5.569719	47.2389	48.63366	18.87499
25245	2	31	80.6	172.5	27.08675	536.97	56.44	6.025316	29.696	36.7046	9.979752
25255	2	21	53.2	159.4	20.93799	701.69	65.22	5.328174	17.4931	32.89673	6.884781

25277	2	22	68.7	159.7	26.93686	629.94	66.43	6.045162	31.7057	45.54049	12.43161
25337	2	33	80.9	171.6	27.4735	562.74	56.27	5.732077	38.3199	46.92053	13.01337
25356	2	25	52.2	167	18.71706	627.59	65.19	5.954535	12.8077	24.34216	4.592384
25387	2	41	77.5	177.6	24.5706	508.75	49.88	5.62037	27.0995	34.38024	8.591626
25390	2	28	61.3	165.3	22.43442	625.91	65.01	5.954032	22.9283	36.9377	8.39124
25392	2	37	69.1	159.4	27.19577	552.23	61.36	6.369542	23.6157	34.13043	9.294461
25418	2	38	83.8	170.9	28.69194	480.39	56.6	6.754066	30.7713	36.2757	10.53566
25462	2	34	59.8	161.4	22.95589	592.6	66.68	6.450254	22.361	37.09141	8.58389
25527	2	24	62.9	160.3	24.47843	609.31	67.59	6.358973	23.5813	36.96227	9.176999
25530	2	33	80.7	167	28.93614	605	67.75	6.419435	36.3402	44.8829	13.0303
25536	2	33	82.6	168.8	28.98913	543.25	62.95	6.642612	33.2294	39.92138	11.66212
25616	2	46	68.7	158.6	27.3118	602.03	59.03	5.620792	29.6946	42.5404	11.80514
25698	2	31	69.4	164.4	25.67768	638.78	74.81	6.713534	26.8872	38.11835	9.948141
25708	2	22	78.8	164.3	29.19114	553.69	63.87	6.612613	33.9211	42.75544	12.56593
25722	2	33	81.4	156.3	33.32011	607.94	73.57	6.937179	37.7825	46.03574	15.46581
25793	2	47	68.2	161.1	26.27806	568.72	63.89	6.439872	25.6983	37.16888	9.901781
25812	2	45	108.6	178.6	34.04606	571.13	61.64	6.186863	49.9894	45.681	15.67166
25869	2	38	94.6	166.4	34.16524	479.45	55.81	6.672853	41.4173	43.84226	14.95806
25885	2	47	62.7	156.7	25.53463	634.26	63.64	5.751826	26.221	41.62169	10.67852
25949	2	21	58.9	165.1	21.60833	704.29	73.15	5.953957	19.5527	32.92842	7.173194
26063	2	44	93.7	165.1	34.37522	453.1	51.62	6.530806	41.8994	44.33414	15.37141
26068	2	32	60.1	169.2	20.99296	620.95	66.91	6.176995	17.1936	28.38778	6.005734
26079	2	38	65.1	173.7	21.57652	529.92	52.15	5.6414	21.7997	32.98547	7.225217
26102	2	30	66.3	165.8	24.11818	535.97	61.45	6.572404	23.1076	34.50657	8.40593
26127	2	34	62.5	164.1	23.20934	548.8	60.3	6.29863	17.3249	27.55539	6.433593
26142	2	49	62.3	167.9	22.09971	605.57	63.22	5.984571	22.2407	35.14266	7.889456
26146	2	40	83.4	161.2	32.0949	459.87	55.98	6.978156	34.8003	40.97667	13.39223
26154	2	25	54	169	18.9069	653.29	69.13	6.066014	15.1252	27.90401	5.295753
26158	2	49	85.7	173.3	28.53538	589.57	59.47	5.782364	38.8487	44.95654	12.93539
26171	2	44	70	159.5	27.51545	532.74	66.83	7.191161	27.5507	39.24031	10.82957
26179	2	39	59.9	161.6	22.9374	654.1	68.75	6.025199	23.7692	39.13328	9.101896
26254	2	44	86.8	164.8	31.95989	456.31	41.13	5.167037	33.7999	38.44703	12.44517
26262	2	36	63.1	165.1	23.14916	545.8	69.34	7.282712	20.829	32.58574	7.641424

26287	2	35	69.4	168.3	24.50142	521.27	59.48	6.541104	22.6857	32.23362	8.009104
26290	2	47	61.3	155.9	25.22135	534.61	56.19	6.025108	23.4065	38.03838	9.630398
26291	2	22	88.1	171.1	30.09372	534.29	53.89	5.781946	35.1281	39.3758	11.99926
26318	2	33	49.3	163.3	18.48735	702.46	68.01	5.550013	16.953	34.23334	6.357322
26327	2	43	57.2	164.3	21.18951	601.64	67.48	6.42956	17.8581	30.87719	6.615461
26338	2	39	80	157.6	32.20902	423.55	53.51	7.242243	31.8886	39.62602	12.83876
26366	2	21	87.4	165.3	31.98643	577.87	68.19	6.764464	39.3802	44.62528	14.41226
26369	2	34	56.2	167.5	20.03119	655.38	71.31	6.23735	19.5362	34.26808	6.963226
26373	2	46	50	157.9	20.05422	640.72	74.02	6.622526	16.5237	32.84671	6.627398
26375	2	36	76.1	149.2	34.1859	461.07	47.81	5.944218	31.2911	41.65831	14.05669
26401	2	31	66	164.3	24.44943	519.94	69.56	7.669185	23.8135	35.83327	8.821615
26500	2	25	71.1	157.3	28.73506	457.76	56.94	7.130541	27.0946	37.83348	10.95028
26509	2	46	68.6	156.5	28.00886	582.78	62.49	6.146795	28.9319	42.01316	11.81268
26519	2	42	59.3	169.7	20.59164	659.6	66.53	5.782022	23.0296	38.40635	7.996919
26525	2	21	69.6	165.3	25.47203	501.26	63.06	7.211636	22.2924	31.87516	8.158515
26539	2	34	77.6	162.7	29.31478	596.44	67.96	6.531749	35.7076	46.05709	13.48918
26546	2	37	80.9	159.4	31.83991	567.03	60.3	6.096129	40.6631	49.60052	16.00383
26547	2	43	80.2	162.4	30.40901	535.96	58.13	6.217429	33.6815	41.73197	12.77084
26552	2	29	65.7	163	24.72807	595.62	71.45	6.876633	25.858	38.85879	9.732395
26564	2	43	64.3	168.6	22.62017	652.37	75.83	6.663309	25.2149	39.08445	8.870378
26572	2	26	50.4	170.4	17.35767	587.24	50.34	4.91406	11.5881	22.83428	3.99092
26602	2	24	62.4	165.1	22.89235	706.21	77.47	6.288435	23.4982	37.03905	8.620659
26696	2	30	65.5	159.3	25.81129	499.18	58.64	6.734101	20.2463	30.57388	7.978369
26725	2	26	56	169	19.60716	658.82	72.15	6.277871	20.6628	36.4577	7.234621
26733	2	47	95.1	167.7	33.81539	539.1	65.33	6.946822	40.4527	42.30051	14.38406
26741	2	48	69.5	178.3	21.86161	573.51	66.76	6.672955	21.4775	30.79004	6.755866
26757	2	44	67.4	163.4	25.24386	629.13	67.79	6.176865	25.9616	37.89013	9.723606
26941	2	43	101.4	161.1	39.07031	516.41	64.5	7.159916	52.6733	50.65875	20.29549
26975	2	34	57.8	163.9	21.51642	759.73	73.96	5.580595	23.6075	40.31983	8.788041
26992	2	33	56.1	161.9	21.40273	522.05	57.45	6.308423	16.9491	29.52141	6.466257
26997	2	28	91.9	179.8	28.42733	612.59	70.77	6.622503	39.9632	43.38544	12.36178
27056	2	24	71.7	160.7	27.76434	589.21	66.82	6.500986	28.0671	38.98555	10.8684
27075	2	27	64.2	169.4	22.37218	623.44	68.17	6.26818	20.4274	31.25568	7.118464

27079	2	43	54	155.8	22.24635	688.65	60.61	5.045319	21.5412	39.90042	8.874318
27103	2	49	66.9	165.4	24.45429	619.32	63.02	5.83319	24.6108	36.13067	8.996109
27176	2	32	65.7	161.5	25.18954	601.45	69.91	6.663197	26.274	39.4976	10.07352
27196	2	22	62.3	160.9	24.06445	538.13	60.74	6.47039	20.9263	33.28736	8.083145
27242	2	28	62	159.4	24.40142	531.83	61.72	6.652669	22.4872	35.98269	8.850315
27301	2	48	79.3	162.7	29.95698	663.55	68.34	5.90397	40.2505	50.4637	15.20534
27343	2	36	97.7	170.9	33.4511	476.54	58.68	7.058844	40.6009	41.49376	13.90118
27344	2	42	58.5	172.3	19.7054	653.34	61.3	5.378536	20.3205	34.41825	6.844848
27354	2	48	89.3	154.9	37.21762	435.28	46.21	6.085694	40.854	45.93873	17.02675
27407	2	46	58.6	172.1	19.78499	557.78	44.58	4.58163	14.7443	24.86861	4.978086
27513	2	44	70.3	155.6	29.03596	541.34	53.08	5.620871	30.8816	43.91294	12.755
27648	2	34	67.2	170	23.2526	676.27	70	5.933634	27.5917	40.56571	9.547301
27667	2	45	65.7	165.3	24.04472	579.74	71.49	7.06895	22.2513	33.60163	8.143473
27710	2	42	61.1	150.5	26.97542	584.16	69.55	6.825087	24.7453	40.31604	10.92496
27714	2	22	58.7	174.4	19.29946	680.86	69.4	5.843116	19.7029	33.46298	6.477944
27726	2	42	90.3	164.6	33.32945	506.47	60.39	6.835246	40.2193	44.43822	14.84482
27772	2	32	81.8	168.8	28.70836	578.75	74.65	7.394038	36.4469	44.40444	12.79133
27779	2	38	59.4	171.7	20.14865	674.1	64.67	5.499477	17.9768	29.75262	6.097781
27798	2	33	72.3	165.1	26.52431	514.13	64.68	7.211738	25.967	35.58883	9.526374
27803	2	46	106.7	172.9	35.69229	534.38	48.73	5.22744	49.6099	46.19236	16.59504
27869	2	41	69.3	163.5	25.92374	519.29	54.67	6.035065	26.2816	37.31825	9.831421
27899	2	23	54.2	159.5	21.30482	649.38	80.19	7.078874	18.8022	34.23823	7.390729
27914	2	34	76.7	165.2	28.10446	513.51	65.51	7.313101	26.686	34.50621	9.778301
27917	2	40	94.3	168.2	33.33187	488.31	55.12	6.470777	41.6934	44.0073	14.73721
27924	2	24	59.1	166.7	21.26749	554.78	60.37	6.237969	18.7549	31.02414	6.749064
27951	2	32	70.2	167.3	25.08102	626.22	62.73	5.742371	27.5808	39.04947	9.854055
27952	2	43	52.7	157	21.38018	568.3	50.92	5.136338	17.8257	33.72088	7.231815
28053	2	25	59.2	162.3	22.47422	534.24	62	6.652703	15.9391	26.42507	6.050994
28068	2	45	50.8	156.2	20.821	577.17	60.87	6.045642	12.0548	23.61852	4.940808
28070	2	43	84.1	158.6	33.4341	472.58	59.78	7.251426	35.1377	41.48856	13.96905
28096	2	45	59.9	152.6	25.72277	527.92	59.4	6.450022	22.8948	38.15895	9.831681
28099	2	34	68.4	167.1	24.49645	627.84	72.53	6.622341	27.5272	39.66844	9.858461
28126	2	38	63	163	23.71184	640.04	81.54	7.303087	23.249	36.6604	8.750423

28159	2	38	104.4	159.5	41.03733	509.42	59.84	6.733773	53.645	51.26076	21.08666
28236	2	40	50.1	163.1	18.83345	728.56	84.04	6.612468	14.462	28.59974	5.436513
28245	2	21	76	163.2	28.5347	468.89	61.48	7.516328	28.4805	37.04708	10.69319
28258	2	35	80.8	155.7	33.32991	480.8	67.23	8.015701	33.3435	41.15628	13.75416
28277	2	38	102.8	159.8	40.25683	444.37	60.95	7.862702	47.4674	45.65314	18.5884
28287	2	31	77.2	157.6	31.08171	379.38	46.31	6.997505	29.5505	37.81283	11.89741
28308	2	34	80.9	158.7	32.12141	431.06	56.75	7.546942	31.0071	38.12087	12.3114
28318	2	36	66.3	160.8	25.64138	530.06	66.12	7.150735	23.6698	35.3555	9.154244
28320	2	27	51.3	163.6	19.16685	815.53	82.26	5.78218	21.2656	41.14908	7.945314
28321	2	49	100.9	174.5	33.13602	474.96	50.93	6.146947	42.6447	42.14932	14.00471
28330	2	47	68.9	162.3	26.15665	547.14	59.34	6.217158	26.025	37.41966	9.879926
28334	2	29	62.8	164.2	23.29235	539.29	60.49	6.429898	22.7284	35.76679	8.429903
28337	2	47	54.3	161	20.94827	586.99	61.59	6.014816	17.5995	32.1391	6.789669
28344	2	36	52.8	157.1	21.39349	624.05	71.54	6.571619	18.625	34.84585	7.546471
28370	2	35	50.5	161	19.48227	524.76	52.29	5.712165	13.5742	26.67759	5.236758
28374	2	31	83.6	161.1	32.21182	591.28	69.67	6.754535	40.6577	48.3577	15.66577
28404	2	29	85.3	166.3	30.84356	484.59	64.14	7.587477	32.4281	38.08824	11.72565
28448	2	28	69.6	166.5	25.10619	513.7	57.26	6.389761	21.3379	30.21415	7.69703
28487	2	47	48.6	152.8	20.81563	759.9	71.56	5.398297	20.467	40.89303	8.766121
28613	2	23	72.8	168.5	25.6408	557.5	69.54	7.150438	22.9693	31.16188	8.089989
28622	2	23	56.8	170	19.65398	665.2	73.91	6.369331	13.9261	24.3311	4.81872
28635	2	44	67.9	168.4	23.94339	490.98	58.02	6.774181	21.1543	30.54481	7.459582
28638	2	40	74.7	167.8	26.52997	571.65	62.71	6.288535	31.1261	41.13574	11.05454
28668	2	25	54.2	162.1	20.62687	505.15	61.85	7.018789	12.3699	22.52148	4.707607
28687	2	41	69.8	164.6	25.76296	560.68	54.98	5.621245	25.9884	36.54147	9.592237
28704	2	29	49.2	158.8	19.51031	651.79	81.3	7.150324	14.5727	29.01489	5.778818
28727	2	41	86.2	173	28.8015	486.53	55.43	6.530976	35.2159	40.66764	11.76648
28748	2	41	73.9	149.5	33.06451	427.96	54.44	7.292187	30.8622	41.546	13.80844
28754	2	21	123.7	172.2	41.71607	450.04	46.67	5.944695	63.2776	50.98649	21.33947
28755	2	38	96.4	164.5	35.62421	540.08	55.43	5.883417	45.5785	46.91806	16.84334
28760	2	42	72.1	163.3	27.03727	547.32	55.4	5.802449	30.6226	41.81011	11.48338
28832	2	21	69.5	160.4	27.0132	499.45	61.06	7.008219	26.295	37.36975	10.22032
28868	2	39	54.8	159.9	21.43303	511.45	65.7	7.363852	15.4178	27.81239	6.030113

28906	2	44	90.6	174.8	29.65141	511.71	57.76	6.470624	37.9165	41.45565	12.40925
28915	2	35	53.8	162.8	20.29895	577.35	75.6	7.506292	16.2977	29.72216	6.149184
28934	2	30	58.8	156.6	23.97694	566.59	55.85	5.650633	22.4383	37.56716	9.149691
28958	2	28	55.3	158	22.1519	560.65	67.75	6.927242	13.4446	24.14151	5.385595
28987	2	42	78.2	158.8	31.01028	498.69	57.52	6.611973	36.4151	46.38541	14.44044
29034	2	25	58.7	170.1	20.28754	612.55	64.06	5.994987	16.7502	28.3109	5.789104
29050	2	46	116.3	173.3	38.72421	552.31	51.72	5.368074	57.5095	49.46309	19.14884
29068	2	39	69.7	168.7	24.49078	559.65	65.65	6.724517	24.6761	35.16046	8.670544
29129	2	31	122.5	166.3	44.29468	439.62	46.67	6.085597	61.9202	50.77199	22.38968
29131	2	38	70.7	165.6	25.78094	634.71	57.54	5.196816	30.857	43.24077	11.25209
29188	2	31	53.9	168.3	19.0292	619.84	60.78	5.621134	14.2437	26.05377	5.028687
29196	2	37	85.8	153.7	36.31947	453.99	49.48	6.247788	39.2807	45.33799	16.62767
29197	2	29	91.7	156.9	37.24976	491.73	58.03	6.765014	45.4033	49.44272	18.44342
29206	2	28	107.2	175.2	34.92421	474.27	49.18	5.944369	48.2822	44.78835	15.72964
29260	2	38	57.6	160.4	22.38792	581.98	58.91	5.802616	16.841	28.96709	6.545746
29263	2	35	59.3	166.2	21.46805	577.65	71.75	7.120328	17.2929	28.93962	6.260453
29277	2	46	55.2	163.6	20.62398	678.24	68.06	5.752431	20.9815	37.67424	7.839167
29317	2	29	61	167.5	21.74204	657.2	71.16	6.206993	21.1074	34.211	7.523243
29332	2	25	51.4	162.4	19.48907	665.23	80.03	6.896422	13.4168	26.01309	5.087178
29344	2	41	60.4	170.2	20.85056	693.02	61.12	5.05569	22.0289	36.05025	7.604553
29347	2	28	70.7	164	26.28644	519.78	53.9	5.944455	28.6939	40.0296	10.66846
29369	2	46	66.2	160.2	25.79485	751.78	75.97	5.792876	29.3153	43.80609	11.42271
29440	2	23	56	162.6	21.18102	571.96	69.42	6.957638	16.7537	29.63452	6.336795
29451	2	39	87.9	167.5	31.32992	467.82	52.97	6.490737	35.3402	39.82066	12.5962
29491	2	35	75.8	169.1	26.50831	530.06	65.55	7.089091	31.5654	41.37701	11.03886
29529	2	34	88.1	166.1	31.93277	552.09	64.37	6.683693	39.5357	44.61332	14.33013
29546	2	37	51.9	163	19.53404	596.2	60.56	5.822865	16.2393	30.99523	6.112123
29590	2	47	67	159.5	26.33622	621.28	67.82	6.257679	31.3199	46.05759	12.31116
29660	2	35	66.4	169	23.24849	616.32	68.15	6.338733	28.8301	42.71695	10.09422
29667	2	24	67.5	169	23.63363	582.48	64.51	6.34876	24.9166	36.5997	8.723994
29680	2	44	68.8	158.4	27.42067	550.1	54.81	5.711642	29.9924	43.20296	11.95366
29735	2	24	84	165.3	30.7421	526.77	53.23	5.792663	36.7479	43.07719	13.4489
29886	2	46	92.3	167.1	33.05589	507.48	51.19	5.782412	40.5836	43.90967	14.53442

29900	2	24	56	168.6	19.7003	669.26	66.56	5.701135	17.4999	30.83025	6.156309
29915	2	45	109.6	169.9	37.96853	468.33	54.44	6.663601	51.8792	47.16445	17.97242
29933	2	33	74.6	169.4	25.99633	565.45	59.23	6.004687	27.5081	36.50957	9.58592
29982	2	21	85.7	168.7	30.11277	527.74	60.97	6.62276	37.9727	43.7817	13.34263
29987	2	27	60.2	157.7	24.20656	594.66	63.87	6.157027	22.8326	37.8608	9.181042
29991	2	39	76.3	165.5	27.85663	453.91	56.7	7.160711	26.4378	34.4132	9.652267
30010	2	27	66.3	173.5	22.02493	620.46	72.56	6.703882	22.213	33.22951	7.379183
30029	2	24	61.4	170.4	21.14605	670.16	79.44	6.795221	19.6859	31.80511	6.779788
30030	2	35	79.4	159.5	31.21039	565.24	68.1	6.906485	32.4935	40.49282	12.77248
30068	2	48	76.3	160.5	29.61928	523.92	60.99	6.673236	30.3753	39.53609	11.79154
30091	2	28	52	171.6	17.65911	661.83	67.11	5.812777	14.3188	27.15159	4.862639
30092	2	36	85.5	176.4	27.47698	610.82	53.76	5.045322	37.8758	43.43794	12.17208
30103	2	31	73.2	180.7	22.41789	678.08	64.93	5.489178	29.2795	40.18956	8.967004
30119	2	46	81.7	161.8	31.20793	437.11	47.79	6.267425	33.7392	40.78842	12.88777
30130	2	39	73.8	163.7	27.53969	613.66	59.95	5.600209	29.0463	38.87254	10.83911
30131	2	26	61.7	162.2	23.45219	628.42	65.72	5.995017	20.9205	33.75047	7.95189
30161	2	42	84.3	154.2	35.45348	499.36	58.48	6.713306	38.5333	45.43609	16.20569
30175	2	42	63.6	160.7	24.62779	666.87	69.86	6.005238	26.8806	41.89084	10.40896
30240	2	24	57.4	152.6	24.6492	660.7	69.44	6.024878	22.3976	38.68605	9.618169
30258	2	41	70.8	165.2	25.94258	567.58	64.97	6.561885	28.0269	39.3392	10.26963
30261	2	34	60.3	173.4	20.05484	581.69	58.57	5.772002	19.6654	31.97189	6.540404
30298	2	36	71.1	173.4	23.64675	495.64	55.07	6.369298	21.8812	30.40027	7.277345
30320	2	27	69.6	158	27.88015	526.11	66.18	7.21096	27.9145	39.61161	11.1819
30336	2	48	52.3	147.6	24.00651	607.54	63.64	6.004795	19.8298	37.88984	9.102184
30360	2	33	61.2	163.5	22.8937	627.22	73.9	6.754099	22.4737	36.28243	8.406962
30363	2	44	82.4	171.6	27.9829	567.14	54.51	5.50971	33.775	40.55722	11.46993
30375	2	26	80.6	168.9	28.25373	654.05	67.01	5.873156	35.7445	44.30756	12.52997
30462	2	49	60.3	166.4	21.77763	601.18	59.9	5.711697	20.2063	32.96404	7.297602
30464	2	35	66.2	179.7	20.50038	565.93	65.98	6.683323	21.8625	32.62062	6.770234
30476	2	43	73.6	166.9	26.42197	605.95	64.97	6.146373	30.6651	41.17608	11.00859
30529	2	48	95.4	175.8	30.86815	495.53	49.46	5.721725	44.0243	45.97353	14.24475
30536	2	40	56.9	161.5	21.8156	518.51	61.28	6.774925	15.4582	26.92489	5.926713
30557	2	28	88.7	179.4	27.55997	576.82	57.78	5.742223	36.6729	41.01976	11.39463

30592	2	44	90	164	33.46222	580.01	59.12	5.84308	46.2424	50.83532	17.19304
30604	2	25	71.2	161.2	27.39996	603.9	73.51	6.977892	28.0763	38.93667	10.80463
30609	2	24	62.6	168.3	22.1007	678.1	74.86	6.328473	23.7376	37.42371	8.380474
30628	2	23	56.6	159.5	22.24821	559.2	57.29	5.872926	17.195	29.9236	6.758974
30645	2	48	46.8	160	18.28125	677.98	60.51	5.116266	12.6124	26.56036	4.926719
30683	2	28	66.8	173.7	22.13996	763.65	72.46	5.439348	24.6178	36.53468	8.159238
30728	2	30	58.2	163	21.90523	633.03	65.42	5.924192	20.3725	34.56909	7.667771
30742	2	34	57.3	169.3	19.99128	639.35	60.32	5.408359	20.2003	34.83801	7.047643
30814	2	23	69	174.7	22.60806	685.36	71.31	5.964507	26.3547	38.10212	8.635197
30819	2	25	61.1	160.2	23.80763	496.93	60.49	6.978004	17.4993	28.28741	6.818607
30828	2	38	73.9	155	30.75963	466.73	48.89	6.004781	30.0329	40.53214	12.50069
30862	2	33	51.9	168.3	18.32311	655.14	64.47	5.641134	12.7388	24.33135	4.497387
30885	2	36	67.1	154.4	28.14673	572.37	60.87	6.096342	28.3519	41.72226	11.89289
30911	2	43	62.5	170.6	21.47445	588.39	61.74	6.015119	20.7005	32.73987	7.112508
30982	2	29	53.9	171.4	18.34709	728.28	79.12	6.227744	14.34	26.01601	4.88121
31029	2	45	85.9	168.9	30.1116	595.09	61.18	5.893451	42.6291	49.43656	14.94331
31037	2	41	126.7	171.9	42.87705	433.63	46.04	6.086377	57.2273	45.20617	19.36652
31045	2	22	59.8	165.2	21.91195	650.54	69.06	6.085488	21.7946	36.49764	7.985991
31051	2	35	62.7	158.9	24.83246	534.65	62.05	6.652962	22.853	36.39813	9.050976
31069	2	46	71.7	163.8	26.72338	553.16	69	7.150579	28.5182	39.39941	10.62905
31070	2	32	85	161.7	32.50865	541.93	62.13	6.572052	39.1471	45.76323	14.97199
31073	2	39	51.5	162.4	19.52698	617.46	60.22	5.590811	15.2559	29.21965	5.784499
31089	2	42	86.5	161	33.37063	562.61	59.93	6.106322	39.862	45.72243	15.37826
31101	2	46	57.5	173.8	19.03568	634.14	55.26	4.995381	18.208	31.55452	6.027854
21177	2	43	76.4	152.7	32.76539	494.88	67.17	7.780693	33.3957	43.60856	14.32229
21205	2	40	50.4	155.8	20.76326	616.67	66.56	6.187331	15.5499	30.64998	6.406085
21233	2	49	78.3	154.1	32.97285	505.6	62.98	7.140662	32.3586	41.14917	13.6265
21274	2	26	57.8	165.4	21.12792	489.23	60.24	7.058538	13.6145	23.23722	4.976577
21316	2	22	89.4	167.8	31.75072	554.54	64.65	6.683108	39.313	43.59982	13.96215
21399	2	42	71.5	163	26.91106	588.56	76.23	7.424685	26.2148	36.57199	9.866687
21514	2	41	94.8	178.1	29.88687	585.77	58.67	5.741585	38.0073	39.65972	11.98227
21730	2	46	83.2	161	32.09753	506.65	57.73	6.531852	31.2383	37.19416	12.05135
21746	2	48	102.4	163.4	38.35269	544.94	54.49	5.732063	46.509	44.96551	17.41939

21777	2	45	91.7	168.8	32.18285	595.45	67.74	6.521429	48.3292	52.21392	16.96152
21819	2	48	83.3	160.8	32.2161	469.8	50.04	6.105864	31.5074	37.72894	12.18542
21866	2	22	68.3	169.1	23.88545	606.24	77.12	7.292313	21.7952	31.55496	7.622082
21935	2	33	97.3	160.4	37.81848	394.33	48.28	7.018597	39.8819	40.1554	15.50126
22012	2	31	79	154.4	33.13847	519.49	66.82	7.373474	34.3247	43.34359	14.39833
22063	2	44	69.6	166.6	25.07606	629.06	62.68	5.711889	26.9295	38.55841	9.70238
22150	2	24	83.2	172	28.12331	574.31	74.9	7.476155	33.3281	39.72848	11.26558
22164	2	23	61.3	167.4	21.87508	688.29	65.18	5.428574	21.5971	34.76523	7.706985
22179	2	35	77.3	151.5	33.67862	530.46	68.14	7.363637	32.1992	41.34256	14.02878
22272	2	48	80.3	165.9	29.1758	544.44	66.08	6.957655	32.6636	40.47675	11.86783
22314	2	46	102.1	159.8	39.98271	504.94	63.16	7.17043	50.1185	48.90798	19.62657
22316	2	41	89.4	170.4	30.7892	507.75	53.72	6.064974	37.8446	42.00247	13.03361
22344	2	30	87	166	31.57207	535.44	60.53	6.480414	39.7509	45.34989	14.4255
22537	2	40	95.3	162	36.31306	516.94	54.79	6.075808	44.6082	46.59306	16.99749
22724	2	48	81.7	159.9	31.95399	548.58	56.69	5.923922	39.366	47.86303	15.39658
22749	2	33	79.3	164.1	29.44801	552.64	66.97	6.946737	29.9547	37.48794	11.12366
22756	2	28	96.3	159.1	38.04398	437.16	63.85	8.372658	43.0602	44.58685	17.01123
22763	2	43	85.8	162.9	32.33293	503.91	64.91	7.384167	35.4372	41.198	13.35418
22790	2	46	66.8	151.4	29.14236	571.07	64.36	6.460551	26.7068	39.606	11.65119
22857	2	46	80.4	152.9	34.3907	453.66	70.23	8.874319	35.9586	44.9906	15.38111
22926	2	36	107.1	179.5	33.23997	528.95	60.45	6.551256	45.5801	42.04186	14.14641
22939	2	32	58.9	161.2	22.66654	635.36	74.3	6.703657	18.0045	30.27814	6.928688
22944	2	22	84	166.9	30.15551	465.31	52.6	6.480167	29.9606	35.16346	10.75568
22969	2	35	127.1	163.5	47.54557	437.72	47.55	6.22726	57.6673	45.21649	21.57218
22979	2	37	66.7	160.3	25.95726	605.64	83.29	7.883538	25.3007	37.35132	9.846128
22989	2	24	55.1	164.5	20.36197	657.85	67.29	5.863629	17.7436	32.03273	6.557072
22998	2	26	88.2	153.5	37.43276	497.66	65.16	7.5057	37.6022	42.3714	15.95866
23120	2	47	76.2	166.2	27.58627	617.66	64.48	5.98437	33.1019	42.9374	11.9837
23139	2	22	59.7	173.9	19.74127	706.94	79.3	6.430333	15.3544	25.49476	5.07731
23164	2	41	81.6	163	30.71248	558.74	64.35	6.602093	33.6031	40.82907	12.64748
23196	2	36	67.7	155.5	27.99806	542.66	58.19	6.147003	28.3771	41.61762	11.73565
23442	2	27	64.6	160.2	25.17141	612.7	68.29	6.389282	26.0745	39.88775	10.15994
23509	2	21	47.4	167.4	16.91482	734.4	83.54	6.520857	12.0546	24.99155	4.301718

23580	2	43	86.3	162	32.88371	447.99	51.68	6.612977	33.8307	39.12687	12.89083
23741	2	26	71.8	157.9	28.79786	488.97	70.9	8.312026	27.4373	37.90421	11.00467
23779	2	48	100.6	171.4	34.24336	473.58	51.11	6.186648	44.3019	43.66079	15.07998
23829	2	37	90.4	166.6	32.57005	481.33	53.05	6.318083	40.5734	44.68343	14.61812
23901	2	30	73.6	166.4	26.58099	517.81	75.36	8.342828	25.0895	33.64376	9.061193
23926	2	31	110.3	173.3	36.7264	446.04	49.4	6.348864	47.8998	43.32698	15.94911
23959	2	44	89.7	168.7	31.51826	537.25	58.55	6.247314	40.4007	44.97507	14.19576
24040	2	31	80.9	162.7	30.56141	487.27	50.01	5.883423	31.4286	38.53688	11.87271
24049	2	23	69	166.5	24.88975	533.55	69.2	7.434878	22.7213	32.49553	8.196052
24079	2	25	75.3	155	31.34235	546	68.01	7.140407	32.7895	43.19466	13.64807
24132	2	48	76.2	166.5	27.48695	517.14	56.45	6.257469	28.7586	37.55872	10.37383
24143	2	37	56	154.1	23.58211	656.26	74.89	6.541702	20.0315	35.32481	8.435449
24157	2	45	90.3	166.7	32.495	467.92	48.77	5.974809	37.4712	41.20205	13.48424
24337	2	46	93.1	176.2	29.98734	590.89	64.3	6.238026	41.1514	43.52361	13.25479
24422	2	22	85.6	161.6	32.77865	488.08	66.6	7.822149	36.3307	42.14977	13.91205
24499	2	32	67.2	169.4	23.4176	624.71	65.55	6.01502	23.3591	34.32905	8.140092
24636	2	22	79.8	167.4	28.47685	671.51	68.68	5.86301	33.6381	41.71453	12.00385
24640	2	46	72.1	169.5	25.0955	492.5	50.63	5.89311	19.5706	26.72258	6.811845
24649	2	33	85.4	163.6	31.90739	579.56	72.19	7.140383	33.2602	38.35046	12.42677
24660	2	44	106.3	171.5	36.1414	485.21	51.6	6.096251	48.5175	45.39683	16.49568
24719	2	31	103.2	171	35.29291	458.73	66.92	8.362606	42.6465	40.7022	14.58449
24725	2	21	79.3	164.3	29.37637	579.72	76.43	7.557679	30.7914	38.36672	11.40655
24897	2	42	68	160.2	26.49622	489.17	62.06	7.272686	22.6178	32.71106	8.813032
24903	2	40	71.2	155.2	29.55946	509.48	70.61	7.944781	24.1806	33.63345	10.03884
24916	2	24	101.9	159	40.30695	415.65	51.92	7.160606	41.7839	41.05541	16.52779
25022	2	34	85.5	169.6	29.72449	483.11	60.6	7.190672	33.1378	38.44979	11.52052
25042	2	49	66.5	152.6	28.557	520.76	64.5	7.100108	22.2819	33.15053	9.568484
25055	2	25	94.2	170.9	32.25275	525.52	55.05	6.004971	40.6871	42.63782	13.93069
25061	2	32	92.3	163.7	34.44327	527.11	64.63	7.028712	42.556	45.86142	15.88047
25106	2	47	101	164.5	37.32412	511.65	62.1	6.957632	46.4611	45.89204	17.1695
25149	2	42	102.1	166.8	36.69726	505.07	60.76	6.896187	44.8249	43.50471	16.11118
25357	2	27	70.6	150.8	31.04574	609.33	75.25	7.079406	31.1575	43.85286	13.70124
25428	2	23	79	164.3	29.26523	425.87	48.07	6.470531	26.2978	33.1353	9.741914

25478	2	46	86.5	163.5	32.35792	459.3	54.93	6.855766	37.9906	43.48139	14.21152
25493	2	24	67.4	170	23.3218	544.56	59.06	6.217139	21.0577	30.76866	7.286401
25556	2	28	113.2	155.9	46.57514	418.39	52.56	7.2014	56.9585	50.1864	23.43507
25745	2	45	67.8	165.2	24.84332	608.87	68.94	6.49067	24.1848	35.6138	8.86181
25802	2	21	72.9	165.5	26.61531	553.81	64.96	6.724006	29.1598	39.50673	10.64605
25982	2	44	94.1	166.7	33.86245	479.82	56.2	6.714301	45.6783	48.16242	16.43761
25985	2	35	111.8	166.2	40.47434	480.29	45.06	5.37812	52.7464	46.89624	19.09549
26039	2	25	89.4	159.9	34.96557	562.06	68.42	6.978197	42.07	46.76247	16.45416
26051	2	48	68.9	150.3	30.5001	647.53	75.27	6.663538	28.6188	41.11985	12.66874
26061	2	31	110.2	161	42.51379	485.19	59.49	7.0287	55.9692	50.61957	21.59222
26167	2	24	86.2	153	36.82344	512.11	61.43	6.876384	39.1773	45.029	16.736
26212	2	30	95	163	35.75596	519.27	60.27	6.65351	43.6259	45.70239	16.41985
26273	2	21	63.9	149.9	28.4379	418.46	52.94	7.252251	20.958	32.51652	9.327099
26473	2	41	100.7	157.6	40.54311	482.34	58.37	6.937121	42.9761	42.54255	17.30273
26493	2	36	80.3	162.4	30.44693	517.4	62.06	6.875879	36.4909	45.19506	13.83606
26502	2	23	75.2	161.9	28.68958	498.36	56.87	6.541584	31.1707	40.73856	11.89194
26574	2	47	50.9	164.7	18.76422	671.25	56.13	4.79351	16.3594	31.7967	6.030873
26770	2	23	91.8	162.1	34.93628	520.09	62.2	6.855746	38.7867	41.78732	14.76103
26857	2	47	62.8	154	26.48001	589.94	57.95	5.631038	24.0767	38.0322	10.15209
26906	2	48	114.8	172.4	38.6249	467.87	55.13	6.754694	51.6655	45.02676	17.38306
26978	2	44	50.8	169.9	17.59855	658.54	50.9	4.430763	12.6766	24.81608	4.391532
26994	2	30	122	169.5	42.46395	465.98	51.44	6.328147	54.6305	44.20931	19.01498
26995	2	30	73.8	161.4	28.33018	551.93	65.81	6.835192	30.7397	41.30498	11.80029
27117	2	30	80.2	158	32.12626	547.55	64.32	6.733876	36.1576	45.238	14.4839
27154	2	46	67.8	161.3	26.05919	493.93	67.66	7.852527	22.9201	33.42891	8.809429
27244	2	39	68.8	166.9	24.69879	632.47	57	5.166278	27.0237	38.16379	9.701349
27269	2	40	84	160.4	32.64905	560.1	63.32	6.480644	39.5207	46.43043	15.36087
27278	2	37	70.8	157.4	28.57747	534.59	61.66	6.611889	30.6725	43.12624	12.38054
27287	2	28	101.3	172.7	33.96446	465.43	58.39	7.191624	38.1325	37.46226	12.78529
27318	2	43	65	164.4	24.0497	546.92	57.39	6.015272	25.2404	38.54161	9.338833
27324	2	45	54.8	168.3	19.34694	691.86	70.89	5.873671	17.6801	31.97483	6.241895
27467	2	28	117.8	159.4	46.36269	444.22	48.81	6.298738	61.4156	52.29735	24.17141
27499	2	23	76	167.5	27.08844	567.03	74.15	7.496318	26.928	34.86511	9.597861

27536	2	35	104.8	161.2	40.33028	591.34	69.05	6.693747	54.403	51.93529	20.93595
27649	2	37	62.4	169.1	21.82214	605.86	69.78	6.602396	23.0539	36.6225	8.062267
27665	2	24	98.5	162	37.53239	468.32	63.57	7.781304	42.5237	43.21694	16.20321
27829	2	37	68.4	163.1	25.71273	633.14	74.04	6.703622	27.1694	39.31805	10.21344
27907	2	34	69	163.6	25.77998	575.81	71.01	7.06941	27.7351	39.93581	10.36247
27925	2	24	61.4	164.8	22.60757	669.4	93.48	8.005268	21.4635	34.54906	7.902893
27971	2	42	85.9	160.2	33.47096	446.98	55.44	7.110137	35.6684	41.23319	13.8982
28015	2	30	87	158.2	34.76212	528.55	48.19	5.226533	42.1199	47.74259	16.82962
28029	2	27	89.2	162.3	33.86319	533.61	56.84	6.106227	41.8285	47.06977	15.87944
28305	2	21	65.7	165.4	24.01565	593.89	79.98	7.720017	20.792	31.51105	7.600204
28352	2	28	54.9	158.6	21.82559	480.92	68.7	8.188922	12.1579	21.86032	4.833394
28418	2	42	91.2	175.6	29.57643	615.96	48.04	4.470883	39.0921	42.48818	12.67769
28462	2	43	52.5	154.5	21.9939	568.18	69.86	7.048318	14.9319	28.11277	6.255443
28526	2	41	64.1	157.3	25.90601	570.14	76.17	7.658528	22.9247	35.48325	9.265017
28565	2	49	71.1	160.5	27.60066	530.19	61.44	6.642974	24.9489	34.63735	9.685038
28615	2	41	100.2	169.9	34.71211	525.34	61.9	6.754497	46.826	46.71398	16.22185
28624	2	42	85	154.9	35.42551	480.17	59.98	7.16068	38.4757	45.15254	16.03554
28713	2	22	59.9	166.2	21.68527	662.9	79.87	6.906826	21.8693	36.1635	7.917222
28730	2	41	78.4	168.8	27.51511	560.5	68.03	6.957732	37.9506	48.32575	13.31907
28813	2	29	76.1	156.4	31.1108	491.74	62.56	7.292964	32.7403	42.62999	13.38472
28853	2	35	101.7	161	39.2346	495.62	61.03	7.058906	43.5083	42.14601	16.78496
28863	2	37	61.9	166.2	22.40932	736.25	80.89	6.298141	22.7141	36.28074	8.223061
28898	2	41	85.1	167.6	30.29574	520.19	56.79	6.258247	36.6483	42.92235	13.04685
29021	2	46	52	168.9	18.22821	694.35	74.94	6.186971	11.5505	22.04365	4.048941
29038	2	43	73.7	160.7	28.5388	588.59	70.81	6.896434	30.5255	41.10116	11.82037
29147	2	44	90.9	156.2	37.25648	476.56	65.96	7.934251	39.946	43.2949	16.37236
29230	2	42	90.5	166	32.84221	469.11	60.93	7.445594	37.8972	41.51389	13.75279
29275	2	45	87.7	164.4	32.4486	531.58	60.1	6.4811	36.433	41.19786	13.48004
29320	2	33	88.4	162.8	33.35366	575.67	66	6.572237	39.4862	44.39385	14.89829
29385	2	43	81.6	155.9	33.5736	492.9	59.74	6.947831	31.3291	38.07546	12.89008
29395	2	45	67.4	165.2	24.69675	618.85	73.03	6.764859	24.0526	35.38875	8.813369
29418	2	21	104.1	159.9	40.71494	440.81	51.32	6.673875	45.8161	43.75335	17.91931
29486	2	34	79.3	168.6	27.89703	505.37	59.64	6.76505	27.8464	34.96381	9.796116

29488	2	37	67.5	163.3	25.31229	539.12	65.05	6.916792	21.8687	31.82068	8.200694
29492	2	33	68.8	160.8	26.60825	638.64	69.49	6.237478	25.6723	37.12928	9.928707
29502	2	37	51.4	165.1	18.85684	661.08	67.5	5.85319	14.1019	26.98939	5.173488
29561	2	23	75.3	153.1	32.12511	587.75	69.88	6.815585	32.0129	42.30884	13.65761
29616	2	36	78.5	177	25.05666	665	72.36	6.237632	28.1864	35.25914	8.996904
29843	2	26	65.9	165.9	23.94378	549.61	65.93	6.876561	19.2058	28.987	6.978139
29847	2	31	53.3	163.2	20.01183	656.31	67.71	5.914073	12.3922	22.97745	4.652733
29866	2	39	104	158.6	41.34538	494.14	60.06	6.967519	54.4618	51.64655	21.65138
29902	2	47	94.4	165.7	34.38167	503.77	55.53	6.318853	40.0933	42.28074	14.60249
29962	2	31	100.8	156.6	41.10333	351.3	58.16	9.4905	41.9078	41.03245	17.08879
30011	2	22	73.1	159.5	28.73399	612.55	76.73	7.180696	29.8801	40.73078	11.74521
30039	2	34	100	173.9	33.06746	543.83	74.59	7.862494	43.0122	43.05659	14.22304
30155	2	48	60.6	153.2	25.81993	567.78	53.97	5.44898	20.9747	34.14307	8.936722
30327	2	41	93.3	156.2	38.24015	392.63	45.22	6.602219	40.2588	43.15511	16.50056
30415	2	46	91.8	157.2	37.14818	464.11	54.03	6.67355	39.9682	43.36027	16.1737
30843	2	39	70.8	171.9	23.95971	664.59	69.26	5.974087	27.6194	38.91109	9.34679
30846	2	45	89.6	165.1	32.87107	567.84	55.98	5.651318	41.0646	45.62419	15.06515
30928	2	28	86.8	156.3	35.53054	518.69	62.58	6.916248	42.2277	48.37644	17.2854
21323	2	28	111	162.9	41.82932	519.76	60.05	6.622973	56.5972	50.77617	21.32813
21576	2	48	56.3	148.5	25.53027	484.34	58.27	6.89664	19.4984	34.24515	8.84191
22392	2	29	50.1	153.2	21.34618	582.67	71.85	7.068821	17.3981	34.45325	7.412834
22754	2	49	74.7	172.1	25.2208	575.45	60.89	6.065704	30.0435	39.97698	10.14352
22848	2	24	70.5	171.6	23.94168	573.31	62.48	6.247329	27.4945	38.54474	9.337084
23028	2	22	45.1	165.2	16.52557	674.21	65.99	5.610813	11.0598	24.24868	4.052539
23359	2	25	45.5	151.9	19.7195	624.07	73.2	6.72389	13.2592	28.92817	5.746478
23485	2	21	52.8	161.6	20.21861	792.54	91.7	6.63271	19.0656	35.6888	7.300755
23577	2	28	114.6	166.8	41.19007	524.27	60.29	6.592242	60.0994	52.4881	21.60121
23756	2	42	63.1	152.2	27.23956	610.45	69.55	6.531154	24.7508	38.58204	10.68464
23790	2	21	69.3	157.7	27.86569	564.69	63.44	6.440149	27.7872	39.8862	11.1733
23880	2	28	50.2	158.5	19.98229	669.81	69.22	5.924106	14.4202	28.30243	5.740011
23941	2	41	101.9	172.8	34.1261	527.89	58.93	6.39935	46.1715	44.78309	15.46274
24289	2	37	58.7	164.2	21.77167	616.29	73.81	6.865512	20.4202	34.17807	7.573797
24291	2	49	82.6	167.1	29.58197	601.74	63.88	6.085537	36.9682	44.25734	13.23962

24357	2	26	76.1	163	28.6424	549.68	58.16	6.06537	31.9093	41.75364	12.00997
24378	2	31	53.5	160.9	20.6653	783.13	72.79	5.328202	20.9414	38.94089	8.088977
24511	2	49	54.8	165.5	20.00712	629.22	58.37	5.317776	18.4997	33.46999	6.754119
24934	2	24	51	158.3	20.35206	553.04	65.06	6.743733	14.6671	28.42406	5.853053
26412	2	48	65	169.2	22.70454	686.03	56.52	4.722826	27.9661	42.46824	9.768574
26513	2	43	78.2	166.8	28.10701	554.11	55.8	5.772728	34.2334	43.35235	12.30433
28197	2	42	55.6	160.6	21.55677	594.21	69.28	6.683605	20.9643	37.30681	8.128105
28325	2	26	72	171.7	24.4226	568.38	60.84	6.136112	24.6183	33.9905	8.350596
28551	2	42	47.2	149.3	21.17495	665.76	67.39	5.802573	17.6338	36.82239	7.910907
28776	2	38	50.7	155.2	21.04866	573.66	56.85	5.680921	16.4262	31.88736	6.819518
30114	2	31	51.1	162.1	19.4471	681.39	73.78	6.207057	15.1379	29.1662	5.761023
30271	2	31	54.6	155.9	22.46469	591.58	65.94	6.389668	19.5773	35.57129	8.054908
30421	2	33	54.5	167.6	19.40209	605.32	64.58	6.115837	17.8556	32.34206	6.356622
30519	2	49	50	155.1	20.78483	680.53	72.85	6.136562	16.9764	33.68554	7.057031
30703	2	34	51.2	163.2	19.22338	680.5	68.28	5.751859	17.1813	33.17782	6.450832
30868	2	35	62.2	160.8	24.05572	584.6	67.23	6.592455	20.5853	32.85133	7.961321

SEQN	original code
sex	1:men; 2: women
age	in years
weight	in kg
height	in cm
BMI	in kg/m^2
R	resistance at 50 kHz
Xc	reactance at 50 kHz
PhA	phase angle
FM	fat mass based on DXA
FM%	
FMI	fat mass index (kg/m^2)
